# Synthesis and Evaluation of Fluorine-Substituted Phenyl Acetate Derivatives as Ultra-Short Recovery Sedative/Hypnotic Agents

**DOI:** 10.1371/journal.pone.0096518

**Published:** 2014-05-05

**Authors:** Heng Zhang, Xiangqing Xu, Yin Chen, Yinli Qiu, Xin Liu, Bi-Feng Liu, Guisen Zhang

**Affiliations:** 1 Systems Biology Theme, Huazhong University of Science and Technology, Wuhan, China; 2 Jiangsu Nhwa Pharmaceutical Co., Ltd., Xuzhou, China; McLean Hospital/Harvard Medical School, United States of America

## Abstract

**Background:**

Soft drugs are molecules that are purposefully designed to be rapidly metabolized (metabolically labile). In anesthesia, the soft drug is useful because it enables precise titration to effect and rapid recovery, which might allow swift and clear-headed recovery of consciousness and early home readiness. Propofol may cause delayed awakening after prolonged infusion. Propanidid and AZD3043 have a different metabolic pathway compared to propofol, resulting in a short-acting clinical profile. Fluorine imparts a variety of properties to certain medicines, including an enhanced absorption rate and improved drug transport across the blood-brain barrier. We hypothesized that the introduction of fluorine to the frame structure of propanidid and AZD3043 would further accelerate the swift and clear-headed recovery of consciousness. To test this hypothesis, we developed a series of fluorine-containing phenyl acetate derivatives.

**Methodology/Principal Findings:**

Fluorine-containing phenyl acetate derivatives were synthesized, and their hypnotic potencies and durations of LORR following bolus or infusion administration were determined in mice, rats and rabbits. The metabolic half-lives in the blood of various species were determined chromatographically. *In vitro* radioligand binding and γ-aminobutyric acid_A_ (GABA_A_) receptor electrophysiology studies were performed. Among the 12 synthesized fluorine-containing phenyl acetate derivatives, compound 5j induced comparable duration of LORR with AZD3043, but more rapid recovery than AZD3043, propanidid and propofol. The time of compound 5j to return to walk and behavioral recovery are approximately reduced by more than 50% compared to AZD3043 in mice and rats and rabbits. The HD_50_ of compound 5j decreased with increasing animal size.

**Conclusions/Significance:**

The rapid recovery might make compound 5j suitable for precise titration and allow swift and clear-headed recovery of consciousness and early home readiness.

## Introduction

Soft drugs are molecules that purposefully designed to be rapidly metabolized (metabolically labile). In anesthesia, the soft drug is useful because it enables precise titration to effect and rapid recovery [1], which might allow swift and clear-headed recovery of consciousness and early home readiness [2].

Propofol is the leading hypnotic drug used for induction and maintenance of general anesthesia. However, propofol has been associated with largely delayed awakening after prolonged infusion [3], which might mean a waste of few days for the complete dissipation of drug effect.

**Figure 1:**
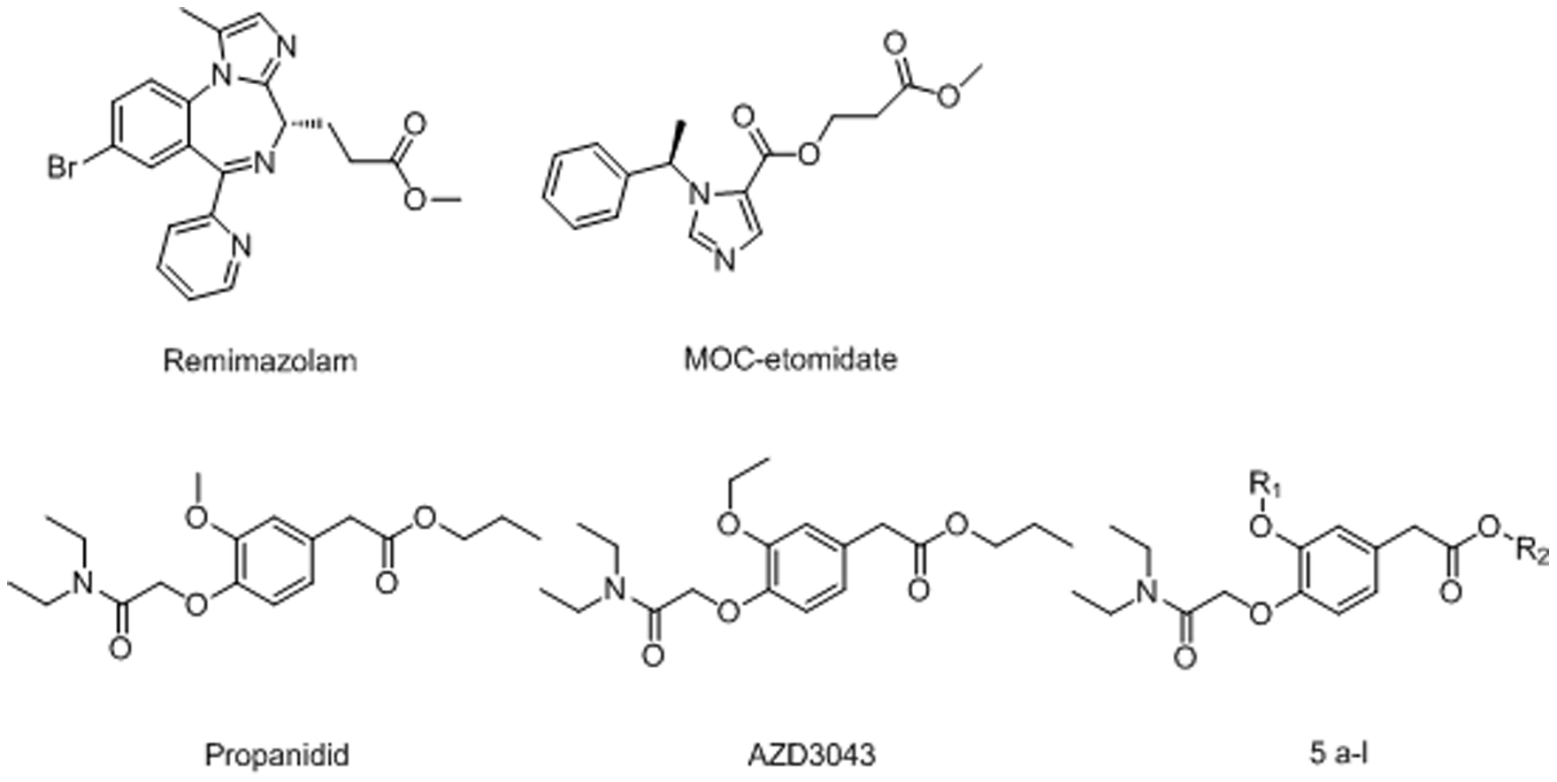
Title and reference compounds.

To resolve this kind of clinical problem, the soft drug is becoming a anesthesia drug discovery trend recently, like remimazolam [4], MOC-etomidate [5] and AZD3043 [6] (Figure 1). A common theme observed within this series of soft drug molecules is the ester structure. Soft drug is metabolically fragile and thus rapidly eliminated, enabling anesthesiologists to manipulate the drug concentration up and down as needed [2].

Propanidid is a non-barbiturate induction agent, formulated as a cremophor EL solution. Unfortunately, anaphylactic reactions caused it to be withdrawn shortly after its introduction [7] [8]. Several cases of negative reactions have been recorded for various drugs using cremophor EL as a solubilizer [9] [10]. Propanidid is hydrolyzed by esterases to an inactive metabolite, resulting in its rapid and predictable recovery regardless of the duration of infusion. Although propanidid was withdrawn, its rapid and predictable recovery was a favorable characteristic for further research. AZD3043 has a very similar structure to that of propanidid, also showed short acting hypnotic profile.

The role of fluorine in medicinal chemistry in recent years has been remarkable [11]. Drug candidates with one or more fluorine atoms have become commonplace [12]. The chief advantage of fluorine, as trifluoromethyl- or fluoro-substituted aryl compounds, is that it imparts a variety of properties to certain medicines, including increased lipid solubility and improved drug transport across the blood-brain barrier [13].

We aimed to introduce fluorine atoms to the ether (R_1_) and the ester (R_2_) residues of the parent structure of propanidid to better meet the demand of precise titration to effect and rapid recovery because of the enhanced absorption rate and transport rate across the blood-brain barrier. The 12 synthesized compounds were formulated in a lipid emulsion similar to that used for propofol to avoid anaphylactic reactions that may be caused by cremophor EL.

## Results and Discussion

### 1. Synthesis of compounds 5 (a-l)

**Figure 2:**
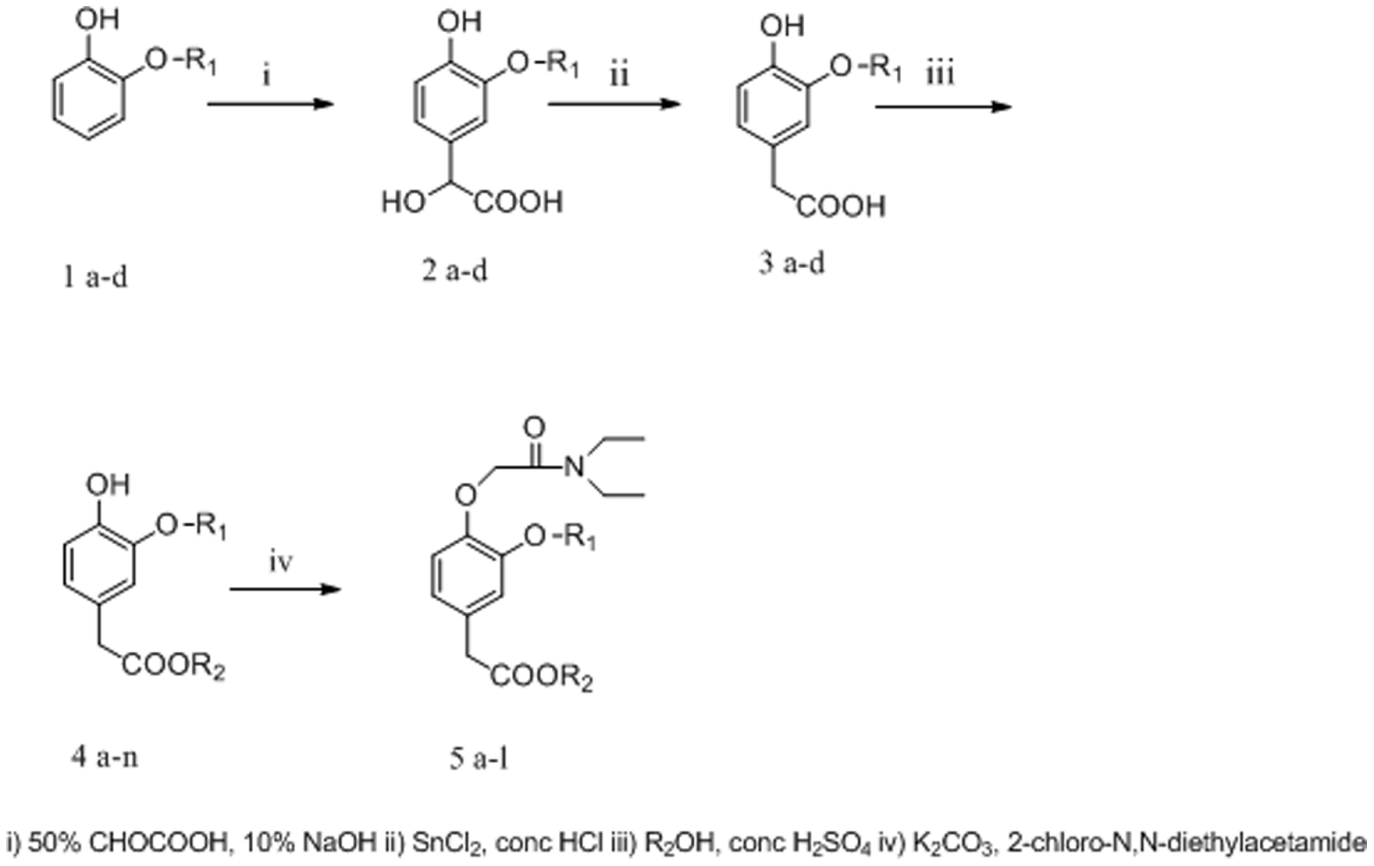
Synthesis of compounds 5a-l.

Fluorine-containing phenyl acetate derivatives were synthesized readily via the following methods (Figure 2).

Compounds 5 (a-l) were prepared by a four-step reaction. Commercially available phenols and glyoxylic acid (50% aqueous solution) were converted to the corresponding 2-OH-phenyl acetic acid derivatives 2 (a-d) in 80–90% overall yield according to previous synthetic procedures used for similar compounds [14]. Intermediate phenyl acetic acid derivatives 3 (a-d) were prepared from the reduction of 2 (a-d) with dihydrate stannous chloride, which were further esterified with various ethanols to afford 4 (a-n). The corresponding esters were reacted with 2-chloro-N, N-diethylacetamide to obtain the final title compounds 5 (a-l); see compounds data in Supporting Information.docx.

### 2. Hypnotic Activity of new compounds

We tested the hypnotic activities of fluorine-containing phenyl acetate derivatives first in mice. Compounds 5a–e (Table 1), with trifluoromethyl or trifluoroethyl group introductions to R_1_ of the parent structure, resulted in reduced anesthetic potency (43.8–72.5 mg/kg) and slower onset times compared to propanidid.

**Table 1:** Hypnotic activities of fluorine substituted phenyl acetate derivatives.

Compd	R_1_	R_2_	Onset time (s) mean ± SD (n = 10)	Duration of LORR (s) mean ± SD (n = 10)	HD_50_ (mg/kg)^a^	LD_50_ (mg/kg)^a^	TI
5a	CF_3_	Et	35.1±6.8	155.2±10.3	72.5 (67.9∼75.5)	441.9 (437.7∼445.3)	6.1
5b	CF_3_	n-Pr	32.4±5.6	162.6±15.7	58.7 (54.4∼63.4)	346.4 (281.8∼387.5)	5.9
5c	CF_3_	CH_2_CF_3_	N.A.^b^	N.A.	N.T.^c^	N.T.	N.T.
5d	CH_2_CF_3_	Et	25.2±3.8	181.9±14.3	47.4 (42.3∼51.3)	260.3 (199.2∼290.4)	5.5
5e	CH_2_CF_3_	*n*-Pr	23.4±4.6	224.6±19.5	43.8 (38.9∼48.2)	249.7 (191.8∼281.8)	5.7
5f	CH_2_CF_3_	CH_2_CF_3_	N.A.	N.A.	N.T.	N.T.	N.T.
5g	Me	CH_2_CF_3_	6.2±0.4	44.8±2.1	59.6 (40.0∼74.7)	362.6 (319.2∼400.8)	6.1
5h	Me	CH_2_CH_2_CF_3_	7.5±1.2	69.3±7.8	25.6 (25.4∼27.8)	N.T.	N.T.
5i	Me	CH(CH_3_)CF_3_	7.2±0.5	80.1±9.3	21.1 (18.8∼23.1)	111.9 (116.2∼119.8)	5.5
5j	Et	CH_2_CF_3_	5.3±1.2	59.1±7.4	24.6 (22.7∼26.2)	192.2 (149.5∼230.1)	7.8
5k	Et	CH_2_CH_2_CF_3_	7.6±0.9	65.6±6.8	29.2 (28.8∼29.6)	N.T.	N.T.
5l	Et	CH(CH_3_)CF_3_	7.3±1.1	121.2±11.5	20.7 (18.2∼22.3)	103.4 (71.8∼116.8)	5.1
Propofol			8.1±1.6	240.9±93.7	11.9 (11.7∼13.5)	64.4 (56.9∼73.3)	5.1
Propanidid			6.7±0.5	89.6±18.2	20.9 (18.6∼23.6)	118.76 (112.0∼124.2)	5.6
AZD3043			5.6±1.2	63.8±8.5	17.6 (16.2∼19.6)	98.4 (105.4∼112.5)	6.0

a 95% confidence limits. ^b^Data not available. ^c^Data not tested. Data was expressed as means ± SD (n = 10).

**Figure 3:**
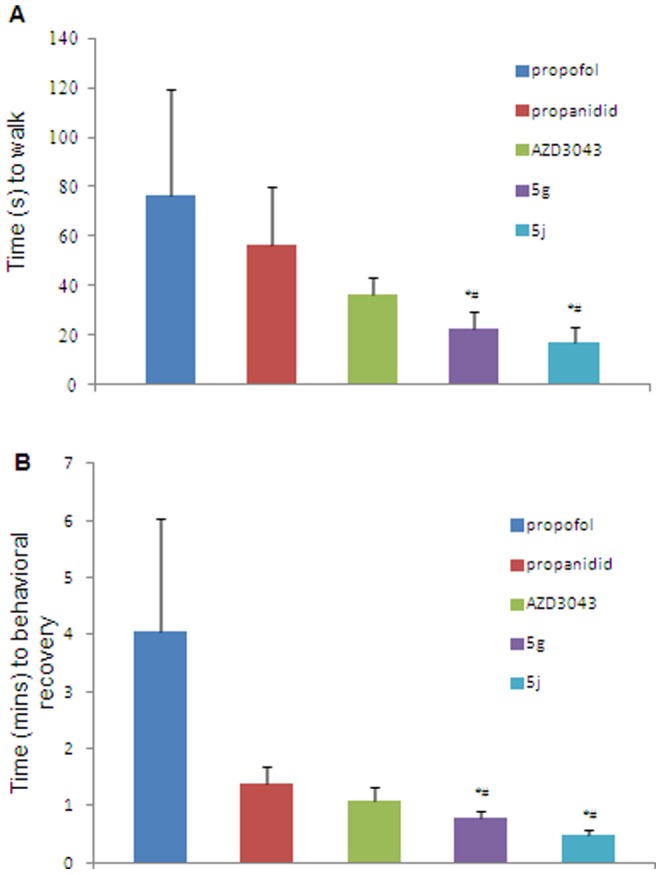
Recovery of propofol, propanidid, AZD3043, 5g and 5j in mice. (A), time to walk. (B), time to behavioral recovery. 2× HD_50_ administration of propofol, propanidid, AZD3043, 5g and 5j in mice. *P<0.05 versus propanidid; ^#^P<0.05 versus AZD3043. a one-way ANOVA with Dunnett's *post hoc* test.

After introducing the trifluoroethyl, trifluropropyl or trifluro-2-propyl group to R_2_ of the parent structure, compounds 5g−l displayed comparable hypnotic potency, onset times and durations of the loss of righting reflex (LORR) compared to propanidid, while compounds 5g (duration of LORR  = 44.8±2.1 s) and 5j (duration of LORR  = 59.1±7.4 s) showed a slightly shorter duration of LORR than propanidid (duration of LORR  = 89.6±18.2 s) and AZD3043 (duration of LORR  = 63.8±8.5 s). However, the times to walk and to behavioral recovery were significantly shortened for 5g (22.5±6.9 s, 46.5±8.5 s, respectively) and 5j (17.3±5.8 s, 28.2±6.4 s, respectively) compared to propofol (76.5±42.5 s, 242.7±119.2 s, respectively), propanidid (56.3±23.5 s, 83.9±17.1 s, respectively) and AZD3043 (36.7±6.4 s, 65.5±14.1 s, respectively) (Figure 3).

However, the introduction of fluorine-containing alkyl groups to both R_1_ and R_2_ yielded unfavorable results. Compounds 5c and 5f displayed poor anesthetic potency.

**Figure 4:**
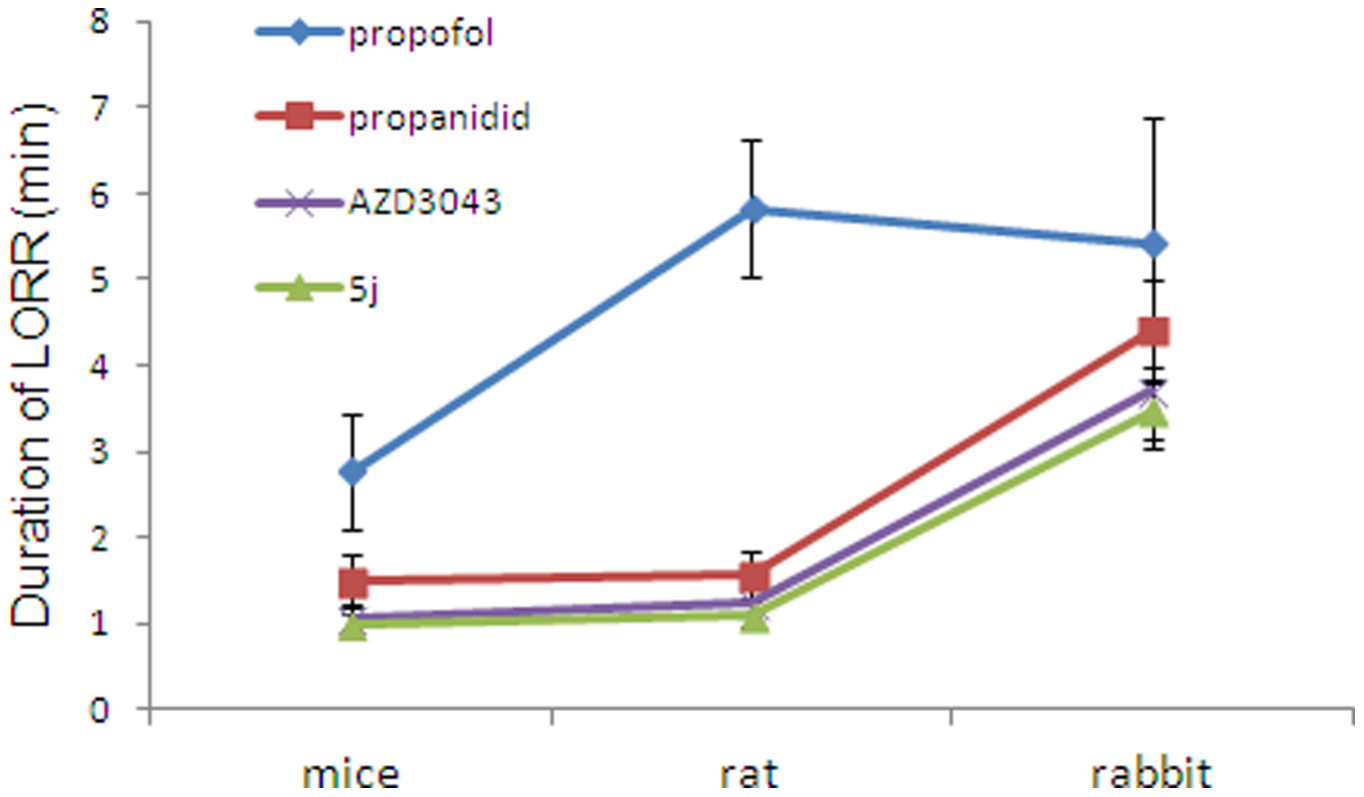
Duration of LORR in mice, rat and rabbit. 2× HD_50_ administration in mice, rat and rabbit (n = 10 in mice, n = 8 in rat, n = 6 in rabbit for each tested compounds).

Based on the property of rapid emergence from hypnosis, we selected compound 5j to further test its hypnotic activity in rats (Table 2) and rabbits (Table 3). The results were consistent with the data in mice. The times to walk and to behavioral recovery were significantly shortened for compound 5j compared to AZD3043, propanidid and propofol in rats and rabbits. Interestingly, when we compared the duration of LORR of compound 5j in mice, rats and rabbits, we found that it increased with increasing animal size (Figure 4); when we compared the HD_50_ value of compound 5j in mice, rat and rabbit, we found that it decreased with increasing animal size.

**Table 2:** Hypnotic potency of propofol, propanidid and 5j in rat.

Compound	HD_50_ (mg/kg)^a^	Duration of LORR (s)	Time (s) to walk	Time (s) to behavioral recovery	TI
propofol	5.2 (4.5∼5.7)	349.3±93.3	286.5±86.5	335.8±156.9	5.9
propanidid	8.4 (7.8∼10.4)	93.3±16.1	58.7±18.8	79.9±34.6	9.6
AZD3043	8.2 (7.7∼9.8)	74.2±10.5	38.4±9.6*	56.9±12.5*	10.1
5j	12.9 (10.6∼13.7)	66.5±8.7	14.2±5.4*^#^	22.8±3.2*^#^	13.3

2× HD_50_ dose of test compound. Data was expressed as means ± SD (n = 10). ^a^95% confidence limits. *P<0.05 versus propanidid; ^#^P<0.05 versus AZD3043; a one-way ANOVA with Dunnett's *post hoc* test.

**Table 3:** Hypnotic potency of propofol, propanidid and 5j in rabbits.

Compound	HD_50_ (mg/kg)^a^	Duration of LORR (s)	Time (s) to walk	TI
propofol	3.2 (2.3∼4.2)	326.6±48.3	425.3±162.3	5.4
propanidid	5.1 (4.7∼5.7)	265.4±44.2	124.5±24.8	14.7
AZD3043	4.8 (4.6∼5.6)	221.6±38.8	89.6 ±7.6*	15.6
5j	6.2 (4.7∼7.0)	208.3±19.8	15.5±7.5*^#^	21.2

2× HD_50_ dose of test compound. Data was expressed as means ± SD (n = 10). ^a^95% confidence limits. *P<0.05 versus propanidid; ^#^P<0.05 versus AZD3043; a one-way ANOVA with Dunnett's *post hoc* test.

Propanidid and AZD3043 were verified to be metabolized to be the corresponded carboxylate metabolites [6] [7]. Compound 5j shared the same carboxylate metabolite with AZD3043. We tested the hypnotic activities of the carboxylates of new derivatives. All the carboxylates of new derivatives (Table 4) didn't show hypnotic potencies with 500 mg/kg administration.

**Table 4:** Chemical data of fluorine substituted phenyl acetate derivatives.

Compound	Octanol:Water Partition (Coefficient ± SD)	GABA_A_ binding (50 µmol) (Mean ± SD)	Metabolites
5a	2157±185	69.1±4.2	2-(4-(2-(diethylamino)-2-oxoethoxy)-3-(trifluoromethoxy)phenyl)acetic acid
5b	4745±280	73.9±5.3	2-(4-(2-(diethylamino)-2-oxoethoxy)-3-(trifluoromethoxy)phenyl)acetic acid
5c	3427±130	30.4±3.5	2-(4-(2-(diethylamino)-2-oxoethoxy)-3-(trifluoromethoxy)phenyl)acetic acid
5d	778±77	67.7±3.2	2-(4-(2-(diethylamino)-2-oxoethoxy)-3-(2,2,2-trifluoroethoxy)phenyl)acetic acid
5e	872±65	72.2±6.5	2-(4-(2-(diethylamino)-2-oxoethoxy)-3-(2,2,2-trifluoroethoxy)phenyl)acetic acid
5f	1452±156	45.4±4.3	2-(4-(2-(diethylamino)-2-oxoethoxy)-3-(2,2,2-trifluoroethoxy)phenyl)acetic acid
5g	597±68	87.2±5.7	2-(4-(2-(diethylamino)-2-oxoethoxy)-3-methoxyphenyl)acetic acid
5h	792±36	80.3±7.6	2-(4-(2-(diethylamino)-2-oxoethoxy)-3-methoxyphenyl)acetic acid
5i	1857±124	81.6±10.5	2-(4-(2-(diethylamino)-2-oxoethoxy)-3-methoxyphenyl)acetic acid
5j	648±36	90.5±6.9	2-(4-(2-(diethylamino)-2-oxoethoxy)-3-ethoxyphenyl)acetic acid
5k	667±102	74.0±6.3	2-(4-(2-(diethylamino)-2-oxoethoxy)-3-ethoxyphenyl)acetic acid
5l	2857±400	79.5±10.0	2-(4-(2-(diethylamino)-2-oxoethoxy)-3-ethoxyphenyl)acetic acid
Propanidid	394±40	70.2±5.8	2-(4-(2-(diethylamino)-2-oxoethoxy)-3-methoxyphenyl)acetic acid
AZD3043	466±54	83.2±7.4	2-(4-(2-(diethylamino)-2-oxoethoxy)-3-ethoxyphenyl)acetic acid

### 3. Hypnosis in Response to IV Infusion

**Figure 5:**
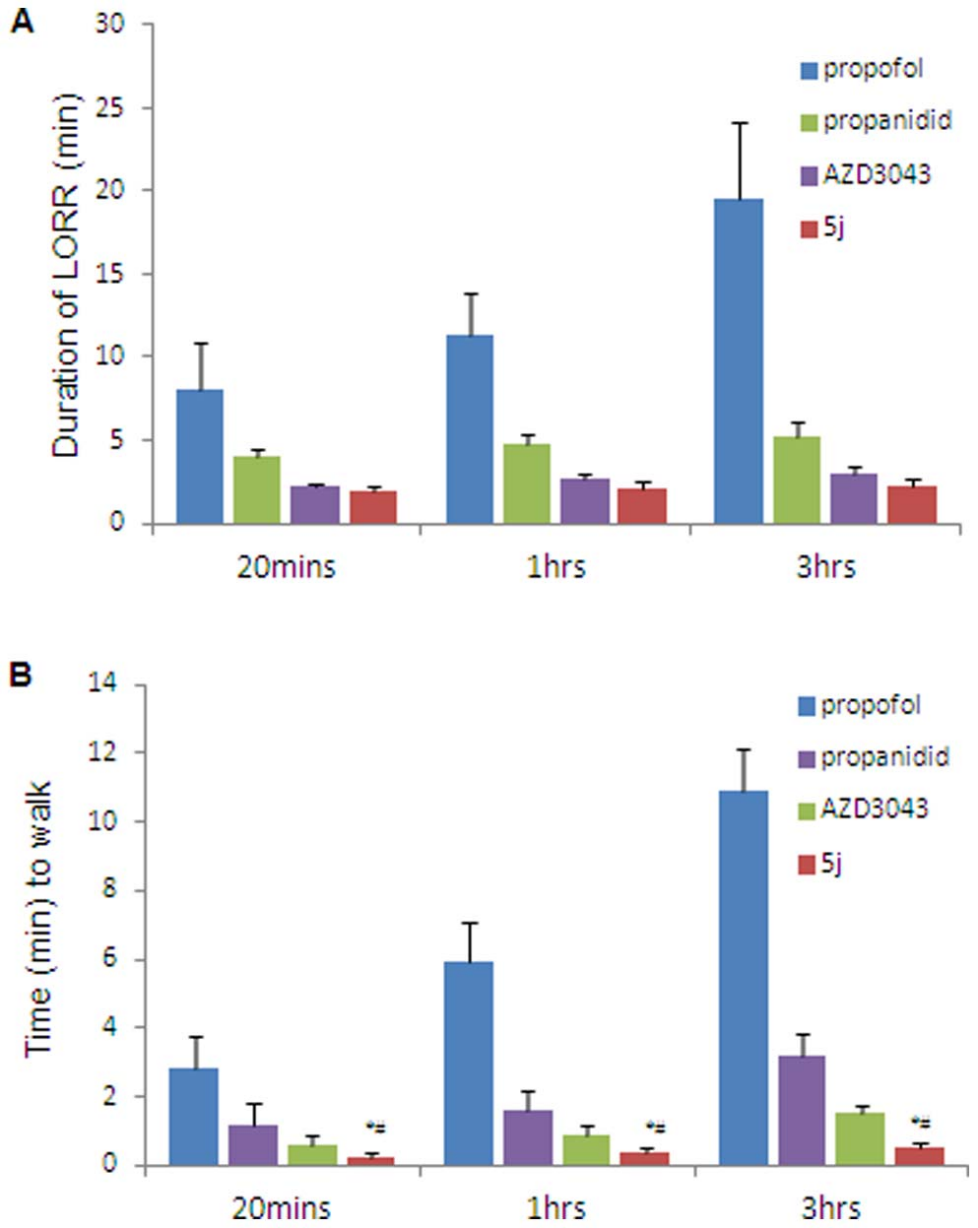
Recovery of propofol, propanidid, AZD3043, 5J in rabbits after a 20-min or 1-h or 3-h IV infusion. (A), Duration of LORR. (B), the time to walk. Induction of hypnosis in rabbits was achieved using 2× HD_50_ dose of test compound, and immediately after induction, infusion via the tail vein was commenced at half the HD_50_ dosage per min. propofol (1.5 mg·kg^−1^·min^−1^; n = 4, 4, or 3, respectively), propanidid (2.5 mg·kg^−1^·min^−1^; n = 5, 4, or 5, respectively), AZD3043 (2.4 mg·kg^−1^·min^−1^; n = 5, 4, or 5, respectively), 5j (3.0 mg·kg^−1^·min^−1^; n = 5, 4, or 5, respectively) in rabbits. *P<0.05 compared with propanidid,^ #^P<0.05 versus AZD3043, a one-way ANOVA with Dunnett's *post hoc* test.

In 20-min, 1-h and 3-h infusion tests in rabbits, the durations of LORR of compound 5j was 117.5±47.2 s, 125.8±57.6 s and 132.9±56.2 s, respectively after the cessation of drug infusions. We compared the duration of LORR and the time to walk for 20-min, 1-h and 3-h infusions with propofol, propanidid, AZD3043 and 5j in rabbits (Figure 5). In 20-min infusion, the duration of LORR was rapid in rabbits treated with 5j (117.5±47.2 s), propanidid (246.6±65.7 s) and AZD3043 (135.6±66.7 s) but the duration of LORR was prolonged and more variable in those that had received propofol (485.6±164.1 s). However, the time to walk was more rapid after 5j infusion (15.5±7.55 s) compared to propofol (167.9±60.8 s), propanidid (70.2±14.9 s) or AZD3043 (46.3±11.2 s). In 1-h or 3-h infusion, the rapid recovery of compound 5j was maintained and was relatively unaffected by the duration of infusions.

### 4. Mechanism of Action: Allosteric Modulation of GABA_A_ Receptors

**Figure 6:**
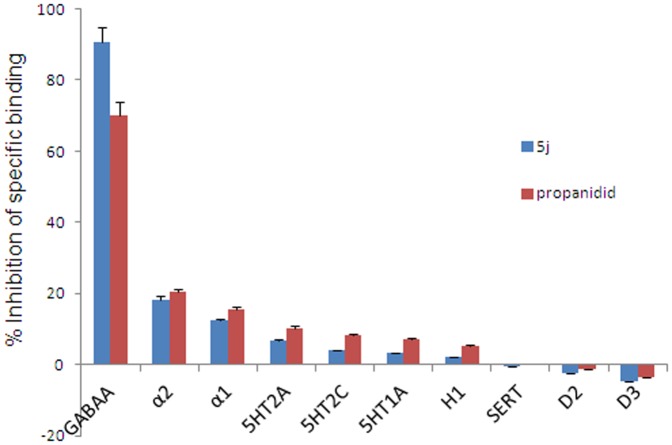
Specific radioligand binding to off-target receptors by compound 5j (50 µmol). Data were expressed as the means ± SD.

In the *in vitro* radioligand-binding test, compound 5j (90.5%) showed better binding affinity than propanidid (70.2%) and AZD3043 (83.2%) (Table 4). To investigate the off-target activities of drug candidate 5j, we tested its binding profile to GABA_A_ and dopamine D_2_, D_3_, H_1_, 5HT_1A_, 5HT_2A_, 5HT_2C_, 5HTT, α_1_ and α_2_ receptors. Compound 5j displayed selective binding to the GABA_A_ receptor (Figure 6).

**Figure 7:**
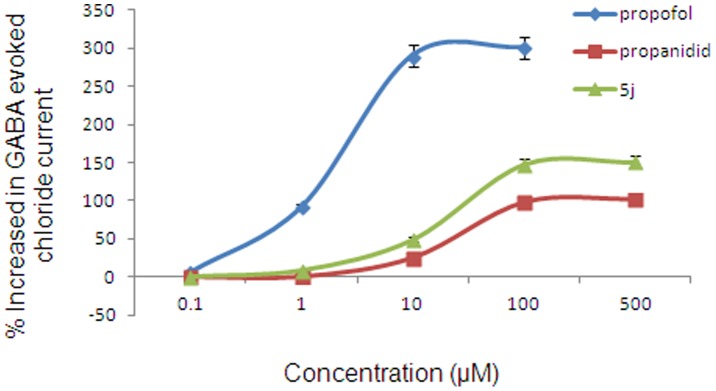
Potentiation of EC_20_ muscimol evoked chloride currents in GABA_A_ (α_1_β_2_γ_2_) CHO-K1 cell. Data were expressed as the means ± SD. EC_50_  =  concentration producing 50% of maximal effect.

We then examined the effect of the selected compound 5j with GABA_A_ receptors using an α_1_β_2_γ_2_-containing CHO K1 cell line. The maximum potentiation achieved by compound 5j (EC_50_ = 12.0 µM) was approximately 50% of that produced by propofol (EC_50_ = 2.4 µM) and 150% of that of propanidid (EC_50_ = 25.2 µM) (Figure 7) (EC_50_ value of AZD3043 was 36 µM [6]).

### 5. Measurement of In Vitro Metabolic Half-lives in Blood

Compound 5j was metabolized rapidly in rat whole blood (mean t_1/2_ = 0.4 min) but more slowly in human blood (mean t_1/2_ = 25.5 min), whereas it was stable in dog and monkey blood (Table 5). The rank order of stability of compound 5j in blood from the various species tested was dog > monkey > human > rabbit > rat.

**Table 5:** The in vitro stability of 5j and AZD3043 in various species.

Preparation	Species	Mean t_1/2_ Value (min) of 5j	Mean t_1/2_ Value (min) of AZD3043
Whole blood	Sprague-Dawley Rat (n = 3)	0.4±0.1	0.6±0.1
	New Zealand White rabbit (n = 3)	11.6±1.6	14.8±3.3
	Beagle dog (n = 3)	>60	>60
	Cynomolgus monkey (n = 3)	48.9±8.8	56.7±11.9
	Human (n = 4)	25.5±5.6*	28.6±6.2

Data are expressed as means ± SD with respect to the mean t_1/2_ values from at least three separate studies.

*Human whole blood stability experiments were conducted in whole blood from four healthy human donors.

## Conclusions

Compound 5j displayed the favorable characteristic of an ultra-short recovery time (the time to walk and the time to behavioral recovery) compared to AZD3043, propanidid and propofol in mice, rats and rabbits. The increased duration of LORR and decreased HD50 value with increasing animal size made us believe that although the recovery times after continuous infusions are shortened by only a few minutes than propanidid and AZD3043 in rabbits, when it comes to human, the infusion dosage would be reduced, and the recovery time would be largely shorten with prolonged infusion time. Of course, it needed to be verified in our undergoing further research.

There was something needed to be mentioned that the infusion strategy was selected based on the infusion pre-experiments. In the pre-experiments, the depth of hypnosis was monitored by the magnitude of a withdrawal reflex to intermittent paw pinch provided by a pair of forceps. However, it was still difficult to make sure the same depths of anesthesia after prolonged infusions with equipotent dose of compound 5j, propanidid, AZD3043 and propofol. These divergent depths of anesthesia might contribute to the different times to return of righting reflexes.

The results of metabolic half-lives of compound 5j in blood from various species showed that it was metabolized faster than AZD3043 in rat, rabbit and human whole blood. The in vitro binding results indicated that compound 5j had a greater binding affinity for the GABAA receptor and a lower affinity for the off-target receptors than propanidid and AZD3043. The electrophysiology study showed that compound 5j had a more potent EC50 against the GABAA receptor compared to propanidid or AZD3043.

Based on the experiment data, compound 5j had comparable duration of LORR with AZD3043 in mice, rats and rabbits, but the recovery time (the time to walk and the time to behavioral recovery) were shorter than AZD3043. It might enable compound 5j for precise titration and rapid recovery, which might allow swift and clear-headed recovery of consciousness and early home readiness.

## Materials and Methods

### Hypnotic Drugs

Propofol was obtained formulated as the emulsion Diprivan (Astra Zeneca). Propanidid and fluorine-containing phenyl acetate derivatives were synthesized readily via a four-step reaction. Melting points were determined in open capillary tubes and are uncorrected. NMR spectra are recorded at 400 MHz on a Varian Inova Unity 200 spectrometer in CDCl_3_ and DMSO-*d*_6_ solution. Chemical shifts are given in δ values (ppm), using tetramethylsilane (TMS) as the internal standard; coupling constants (J) were given in Hz. Signal multiplicities are characterized as s (singlet), d (doublet), t (triplet), q (quartet), m (multiplet), br (broad signal). Reagents were all of analytical grade or of chemical purity. HRMS experiments were performed with TripleTOF 5600 (AB SCIEX). Analytical TLC was performed on silica gel GF254. Column chromatographic purification was carried out using silica gel. Compound purity is determined by high performance liquid chromatography (HPLC), and all final test compounds were >95% purity. HPLC methods used the following: Agilent 1100 spectrometer; column, Shim-pack ODS 5.0 µm×150 mm×2.0 mm I.D (SHIMADZU, Japanese); mobile phase, 0.0167% HCOOH (TEDIA Company,USA)/acetonitrile (Merck Company, Germany) 50/50; flow rate, 0.2 mL/min; column temperature, 40 °C; UV detection was performed at 254 nm.

#### Step 1: General procedure for the synthesis of 2-Hydroxyphenylacetic Acid Derivatives 2 (a-d)

A 10% sodium hydroxide solution (300 ml) was added slowly to the rapidly stirring solution of the appropriate phenol (1a–d) (0.4 mol), 50% glyoxylic acid (0.396 mol) and 100-ml distilled water at 0°C. The reaction mixture was stirred at room temperature overnight and washed three times with ethyl acetate. The aqueous phase was adjusted to pH 3 using concentrated hydrochloric acid and extracted three times using ethyl acetate. The combined organic phases were evaporated under reduced pressure to obtain the final products 2(a-d).

2-hydroxy-2-(4-hydroxy-3-methoxyphenyl)acetic acid (2a): This was synthesized from 1a. Yield: 90%; mp 129–131.5°C. ^1^H-NMR (DMSO-*d*_6_) δ, 3.75 (s, 3H), 4.89 (s, 1H), 5.66 (br s, 1H), 6.71–6.96 (m, 3H), 8.92 (s, 1H), 12.42 (br s, 1H). ^13^C-NMR (DMSO-*d*_6_) δ 56.04, 72.68, 111.29, 115.49, 119.80, 131.59, 146.60, 147.73, 174.84.

2-(3-ethoxy-4-hydroxyphenyl)-2-hydroxyacetic acid (2b): This was synthesized from 1b. Yield: 87%; mp 118–121°C. ^1^H-NMR (DMSO-*d*_6_)) δ 1.32(t, 3H, J = 7.07 Hz), 3.99 (q, 2H), 4.87 (s, 1H), 5.66 (br s, 1H), 6.72–6.94 (m, 3H), 8.84 (s, 1H), 12.42 (br s, 1H). ^13^C-NMR (DMSO-*d*_6_)) δ 15.23, 64.36, 72.65, 112.74, 115.57, 119.86, 131.55, 146.80, 146.91,174.85.

2-hydroxy-2-(4-hydroxy-3-(trifluoromethoxy)phenyl)acetic acid (2c): This was synthesized from 1c. Yield: 85%; mp 155–157.9°C. ^1^H-NMR (DMSO-*d*_6_)) δ 4.97 (s, 1H), 5.66 (br s, 1H), 6.99–7.28 (m, 3H), 10.19 (s, 1H), 12.64 (br s, 1H). ^13^C-NMR (DMSO-*d*_6_)) δ 71.76, 117.71, 120.84 (q, 1C, J = 255.90 Hz), 121.43, 127.12, 132.01, 136.12, 149.68, 174.40.

2-hydroxy-2-(4-hydroxy-3-(2,2,2-trifluoroethoxy)phenyl)acetic acid (2d): This was synthesized from 1d. Yield: 83%; mp 89–91°C.^1^H-NMR (DMSO-*d*_6_)) δ 4.62 (m, 2H), 4.89 (s, 1H) 5.70 (br s, 1H), 6.82–7.06 (m, 3H), 8.49 (s, 1H), 12.48 (br s, 1H). ^13^C-NMR (DMSO-*d*_6_)) δ 66.85 (q, 1C, *J* = 33.39 Hz), 72.34, 116.0, 116.57, 122.26, 123.76 (q, 1C, *J* = 277.95 Hz), 131.73, 145.49, 147.53, 174.66.

#### Step 2: General procedure for the synthesis of phenylacetic acid derivatives 3 (a-d)

2-Hydroxyphenylacetic acid (0.2 mol) was added to a solution of concentrated hydrochloric acid (120 ml) and dihydrate stannous chloride (0.4 mol). After refluxing for 4 h, the reaction was cooled to room temperature. The resulting solid was collected by filtration and washed with distilled water. The white solid was air dried to obtain the final compound 3(a-d).

2-(4-hydroxy-3-methoxyphenyl)acetic acid (3a): This was synthesized from 2a. Yield: 75%; mp 141–142°C. ^1^H-NMR (DMSO-*d*_6_)) δ 3.42 (s, 2H), 3.74 (s, 3H), 6.64–6.81 (m, 3H), 8.82 (s, 1H), 12.18 (s, 1H). ^13^C-NMR (DMSO-*d*_6_)) δ 40.72, 56.04, 114.02, 115.70, 122.08, 126.14, 145.72, 147.76, 173.47.

2-(3-ethoxy-4-hydroxyphenyl)acetic acid (3b): This was synthesized from 2b. Yield: 80%; mp 73–75°C. ^1^H-NMR (DMSO-*d*_6_)) δ 1.31 (t, 3H, J = 7.04 Hz), 3.20–3.41 (m, 3H), 3.99 (s, 2H), 6.63–6.84 (m, 3H), 12.17 (s, 1H). ^13^C-NMR (DMSO-*d*_6_)) δ 15.26, 40.69, 64.35, 115.47, 115.78, 122.3, 126.2, 146.04, 146.84, 173.48.

2-(4-hydroxy-3-(trifluoromethoxy)phenyl)acetic acid (3c): This was synthesized from 2c. Yield: 86%; mp 68–70°C. ^1^H-NMR (DMSO-*d*_6_)) δ 3.50 (s, 2H), 6.95–7.15 (m, 3H), 10.04(s, 1H), 12.32 (s, 1H). ^13^C-NMR (DMSO-*d*_6_)) δ 39.70, 117.77, 120.86 (q, 1C, J = 252.44 Hz), 124.22, 126.65, 129.80, 136.08, 148.85, 173.48.

2-(4-hydroxy-3-(2,2,2-trifluoroethoxy)phenyl)acetic acid (3d): This was synthesized from 2d. Yield: 79%; mp 144–145°C. ^1^H-NMR (DMSO-*d*_6_)) δ 3.42 (s, 2H), 4.58–4.66 (m, 2H), 6.78–6.92 (m, 3H), 9.34 (s, 1H), 12.21 (s, 1H). ^13^C-NMR (DMSO-*d*_6_)) δ 66.79 (q, 1C, *J* = 35.66 Hz), 66.80, 116.72, 118.39, 123.10 (q, 1C, *J* = 277.93 Hz), 124.66, 126.30, 145.49, 146.59, 173.29.

#### Step 3: General procedure for the synthesis of phenylacetic ester derivatives 4 (a-n)

Concentrated sulfuric acid (5 ml) was added to the solution of the compounds 3a–d (0.1 mol) and various alcohols (100 ml). The reaction mixture was refluxed for 3 h, ethyl acetate (300 ml) was added and then washed three times with distilled water and twice with saturated sodium bicarbonate. The organic phase was dried using anhydrous magnesium sulfate and evaporated under reduced pressure. The crude product was purified using chromatography (5% petroleum ether/ethyl acetate) to yield the final compound 4 (a-n).

ethyl 2-(4-hydroxy-3-(trifluoromethoxy)phenyl)acetate (4a): This was synthesized from 3c and ethanol. light yellow oil; yield: 77.4%. ^1^H-NMR (CDCl_3_) δ 1.27 (t, 3H, J = 7.20 Hz), 3.56 (s, 2H), 4.16–4.21 (m, 2H), 6.27 (s, 1H), 6.91–7.16 (m, 3H). MS (ESI) m/z 265.1([M+H]^+^).

propyl 2-(4-hydroxy-3-(trifluoromethoxy)phenyl)acetate (4b): This was synthesized from 3c and n-propanol. light yellow oil; yield: 75.9%. ^1^H-NMR (CDCl_3_) δ 0.92 (t, 3H, J = 7.57 Hz), 1.63–1.68 (m, 2H), 3.57 (s, 2H),4.08 (t, 3H, J = 6.60 Hz), 6.93–7.17 (m, 3H). MS (ESI) m/z 279.1([M+H]^+^).

2,2,2-trifluoroethyl 2-(4-hydroxy-3-(trifluoromethoxy)phenyl)acetate (4c): This was synthesized from 3c and 2,2,2-trifluoroethanol. light yellow oil; yield: 72.5%. ^1^H-NMR (CDCl_3_) δ 3.68 (s, 2H),4.47–4.54 (m, 2H), 5.94 (s, 1H), 6.97–7.19 (m, 3H). MS (ESI) m/z 319.1([M+H]^+^).

ethyl 2-(4-hydroxy-3-(2,2,2-trifluoroethoxy)phenyl)acetate (4d): This was synthesized from 3d and ethanol. light yellow oil; yield: 82.6%. ^1^H-NMR (CDCl_3_) δ 1.27 (t, 3H, J = 7.26 Hz), 3.54 (s, 2H), 4.14–4.19 (m, 2H), 4.40–4.47 (m, 2H), 5.49 (s, 1H) 6.85–6.95 (m, 3H). MS (ESI) m/z 279.1([M+H]^+^).

propyl 2-(4-hydroxy-3-(2,2,2-trifluoroethoxy)phenyl)acetate (4e): This was synthesized from 3d and ethanol n-propanol. light yellow oil; yield: 84.2%. ^1^H-NMR (CDCl_3_) δ 0.93 (t, 3H, J = 7.41 Hz), 1.62–1.71 (m, 2H), 3.55 (s, 2H), 4.07 (t, 2H, J = 6.78 Hz), 4.40–4.46 (m, 2H), 5.50 (s, 1H) 6.86–6.95 (m, 3H). MS (ESI) m/z 293.1([M+H]^+^).

2,2,2-trifluoroethyl 2-(4-hydroxy-3-(2,2,2-trifluoroethoxy)phenyl)acetate (4f): This was synthesized from 3d and 2,2,2-trifluoroethanol. light yellow oil; yield: 73.5%. ^1^H-NMR (CDCl_3_) δ 3.67 (s, 2H), 4.40–4.46 (m, 2H), 4.47–4.53 (m, 2H), 5.51 (s, 1H) 6.85–6.97 (m, 3H). MS (ESI) m/z 333.1([M+H]^+^).

2,2,2-trifluoroethyl 2-(4-hydroxy-3-methoxyphenyl)acetate (4g): This was synthesized from 3a and 2,2,2-trifluoroethanol. light yellow oil; yield: 79.6%. ^1^H-NMR (CDCl_3_) δ 3.67 (s, 2H), 3.90 (s, 3H), 4.47–4.53 (m, 2H) 5.63 (s, 1H), 6.78–6.90 (m, 3H). MS (ESI) m/z 265.1 ([M+H]^+^).

3,3,3-trifluoropropyl 2-(4-hydroxy-3-methoxyphenyl)acetate (4h): This was synthesized from 3a and 2,2,2-trifluoropropanol. light yellow oil; yield: 79.2%. ^1^H-NMR (CDCl_3_) δ 2.04–2.11 (m, 2H), 3.69 (s, 2H), 3.88 (s, 3H), 4.12–4.14 (m, 2H), 5.65 (s, 1H), 6.77–6.90 (m, 3H). MS (ESI) m/z 278.1 ([M+H]^+^).

1,1,1-trifluoropropan-2-yl 2-(4-hydroxy-3-methoxyphenyl)acetate (4i): This was synthesized from 3a and 1,1,1-trifluoro-2-propanol. light yellow oil; yield: 78.6%. ^1^H-NMR (CDCl_3_) δ 1.40 (d, 3H, J = 6.79 Hz), 3.62 (s, 2H), 3.90 (s, 3H), 5.27–5.37 (m, 1H), 5.65(s, 1H), 6.77–6.90 (m, 3H). MS (ESI) m/z 278.1 ([M+H]^+^).

2,2,2-trifluoroethyl 2-(3-ethoxy-4-hydroxyphenyl)acetate (4j): This was synthesized from 3b and 2,2,2-trifluoroethanol. light yellow oil; yield: 81.3%. ^1^H-NMR (CDCl_3_) δ 1.46 (t, 3H, J = 7.04 Hz), 3.65 (s, 2H), 4.10–4.15 (m, 2H), 4.46–4.52 (m, 2H), 5.63 (s, 1H), 6.77–6.90 (m, 3H). MS (ESI) m/z 279.2 ([M+H]^+^).

3,3,3-trifluoropropyl 2-(3-ethoxy-4-hydroxyphenyl)acetate (4k): This was synthesized from 3a and 2,2,2-trifluoropropanol. light yellow oil; yield: 82.6%. ^1^H-NMR (CDCl_3_) δ 1.24 (t, 3H, J = 7.12 Hz), 2.05–2.12 (m, 2H), 3.72 (s, 2H), 4.09–4.15 (m, 2H), 4.45–4.51 (m, 2H), 5.65 (s, 1H), 6.77–6.90 (m, 3H). MS (ESI) m/z 293.1 ([M+H]^+^).

1,1,1-trifluoropropan-2-yl 2-(3-ethoxy-4-hydroxyphenyl)acetate (4l): This was synthesized from 3b and 1,1,1-trifluoro-2-propanol. light yellow oil; yield: 74.5%. ^1^H-NMR (CDCl_3_) δ 1.40 (d, 3H, J = 6.40 Hz), 1.46 (t, 3H, J = 6.98 Hz), 3.61 (s, 2H), 4.10–4.15 (m, 2H), 5.26–5.36 (m, 1H), 5.64(s, 1H), 6.76–6.90 (m, 3H). MS (ESI) m/z 293.1 ([M+H]^+^).

propyl 2-(4-hydroxy-3-methoxyphenyl)acetate (4m): This was synthesized from 3a and n-propanol. light yellow oil; yield: 92.5%. ^1^H-NMR (CDCl_3_) δ 0.93 (t, 3H, *J* = 7.58 Hz), 1.62–1.71 (m, 2H), 3.56 (m, 2H), 3.89 (s, 3H), 4.07 (t, 2H, *J* = 6.77 Hz), 5.62 (s, 1H), 6.80–6.88 (m, 3H). MS (ESI) m/z 225.2 ([M+H]^+^).

propyl 2-(3-ethoxy-4-hydroxyphenyl)acetate (4n): This was synthesized from 3b and n-propanol. light yellow oil; yield: 84.3%. ^1^H-NMR (CDCl_3_) δ 0.93 (t, 3H, J = 7.36 Hz), 1.45 (t, 3H,J = 6.94 Hz), 1.61–1.70 (m, 4H), 3.54 (s, 2H), 4.06 (t, 2H, J = 6.65 Hz), 4.10–4.15 (m, 2H), 5.65 (s, 1H), 6.76–6.88 (m, 3H). MS (ESI) m/z 239.2 ([M+H]^+^).

#### Step 4: General procedure for the synthesis of fluorine-substituted phenyl acetate derivatives 5 (a-n)

Compounds 4a–n (1 mmol), 2-chloro-N,N-diethylacetamide (1 mmol) and K_2_CO_3_ (1.5 mmol) were added to 50-ml acetone. The reaction mixture was refluxed overnight and the acetone was evaporated. Ethyl acetate (300 ml) was added to the residue and then washed three times with distilled water. The organic phase was dried using anhydrous magnesium sulfate and evaporated under reduced pressure. The crude product was purified using chromatography (5–10% petroleum ether/ethyl acetate) to yield the final compound 5 (a-l).

Ethyl 2-(4-(2-(diethylamino)-2-oxoethoxy)-3-(trifluoromethoxy)phenyl)acetate (5a): This was synthesized from 4a and 2-chloro-N,N-diethylacetamide. light yellow oil; yeild 85.8%. ^1^H-NMR (CDCl_3_) δ 1.14 (t, 3H, J = 7.14 Hz), 1.22 (t, 3H, J = 7.30 Hz), 3.39–3.46 (m, 4H), 3.56 (s, 2H), 3.88 (s, 3H), 4.13–4.19 (m, 2H, J = 7.16 Hz), 4.73 (s, 2H), 7.03–7.28 (m, 3H). ^13^C-NMR (CDCl_3_) δ 12.68, 14.09, 14.20, 40.21, 40.26, 41.49, 61.01, 68.95, 114.76, 120.61 (q, 1C, J = 256.58 Hz), 123.81, 127.82, 128.64, 138.01, 149.57, 166.42, 171.05. HRMS (ESI) m/z 378.1521 ([M+H]^+^).

propyl 2-(4-(2-(diethylamino)-2-oxoethoxy)-3-(trifluoromethoxy)phenyl)acetate (5b):

This was synthesized from 4b and 2-chloro-N,N-diethylacetamide. light yellow oil; yeild 87.6%. ^1^H-NMR (CDCl_3_) δ 0.92 (t, 3H, J = 7.30Hz), 1.14 (t, 3H, J = 7.30 Hz), 1.22 (t, 3H, J = 7.04 Hz), 1.62–1.68(m, 2H), 3.39–3.47 (m, 4H), 3.57 (s, 2H), 4.07 (t, 2H, J = 6.65 Hz), 4.73 (s, 2H), 7.03–7.28 (m, 3H). ^13^C-NMR (CDCl_3_) δ 10.26, 12.68, 14.21, 21.88, 40.21, 40.31, 41.50, 66.62, 68.98, 114.75, 120.61 (q, 1C, J = 256.58 Hz), 123.81, 127.87, 128.64, 138.0, 149.57, 166.42, 171.12. HRMS (ESI) m/z 392.1682 ([M+H]^+^).

2,2,2-trifluoroethyl 2-(4-(2-(diethylamino)-2-oxoethoxy)-3-(trifluoromethoxy)phenyl)acetate (5c): This was synthesized from 4c and 2-chloro-N,N-diethylacetamide. light yellow oil; yeild 86.3%. ^1^H-NMR (CDCl_3_) δ 1.14 (t, 3H, J = 7.14 Hz), 1.22 (t, 3H, *J* = 7.14 Hz), 3.39–3.46 (m, 4H), 3.69 (s, 2H), 4.47–4.53 (m, 2H), 4.75 (s, 2H), 7.05–7.28 (m, 3H). ^13^C-NMR (CDCl_3_) δ 12.68, 14.20, 39.43, 40.23, 41.50, 60.69 (q, 1C, *J* = 36.67 Hz), 68.87, 114.89, 120.62 (q, 1C, *J* = 257.54 Hz), 122.79 (q, 1C, *J* = 277.67 Hz), 123.84, 126.29, 128.70, 138.09, 149.94, 166.32, 169.46. HRMS (ESI) m/z 432.2377 ([M+H]^+^).

ethyl 2-(4-(2-(diethylamino)-2-oxoethoxy)-3-(2,2,2-trifluoroethoxy)phenyl)acetate (5d): This was synthesized from 4d and 2-chloro-N,N-diethylacetamide. light yellow oil; yeild 87.6%. ^1^H-NMR (CDCl_3_) δ 1.15 (t, 3H, J = 7.07 Hz), 1.22 (t, 3H, J = 7.07 Hz), 1.26 (t, 3H, J = 7.18 Hz), 3.39–3.43 (m, 4H), 3.54 (s, 2H), 4.13–4.19 (m, 2H), 4.42–4.48 (m, 2H), 4.73 (s, 2H), 6.94–6.95 (m, 3H). ^13^C-NMR (CDCl_3_) δ 12.75, 14.14, 14.20, 40.22, 40.58, 41.43, 60.93, 67.81 (q, J = 35.59 Hz, 1C), 68.48, 115.10, 118.51, 123.50 (q, J = 276.92 Hz, 1C), 124.69, 128.17, 147.25, 147.95, 166.63, 171.41. 19F-NMR (CDCl_3_) δ -74.11 (t, 3F, J = 7.09 Hz). MS (ESI) m/z 392.1682 ([M+H]^+^).

propyl 2-(4-(2-(diethylamino)-2-oxoethoxy)-3-(2,2,2-trifluoroethoxy)phenyl)acetate (5e): This was synthesized from 4e and 2-chloro-N,N-diethylacetamide. light yellow oil; yeild 79.6%. ^1^H-NMR (CDCl_3_) δ 0.92 (t, 3H, J = 7.46 Hz), 1.14 (t, 3H, J = 7.10 Hz), 1.22 (t, 3H, J = 7.10 Hz), 1.61–1.69 (m, 2H), 3.38–3.44 (m, 4H), 3.54 (s 2H), 4.06 (t, 2H, J = 6.78 Hz), 4.42–4.48 (m, 2H), 4.73 (s, 2H), 6.94–6.95 (m, 3H). ^13^C-NMR (CDCl_3_) δ 5.55, 8.0, 9.45, 17.16, 35.48, 35.84, 36.68, 61.79, 63.08 (q, 1C, J = 35.18 Hz), 63.76, 110.35, 113.76, 118.73 (q, 1C, J = 278.42 Hz), 119.94, 123.48, 142.50, 143.19, 161.88, 166.73. HRMS (ESI) m/z 406.1852 ([M+H]^+^).

2,2,2-trifluoroethyl 2-(4-(2-(diethylamino)-2-oxoethoxy)-3-(2,2,2-trifluoroethoxy)phenyl)acetate (5f): This was synthesized from 4f and 2-chloro-N,N-diethylacetamide. light yellow oil; yeild 81.7%. ^1^H-NMR (CDCl_3_) δ 1.13 (t, 3H, J = 7.06 Hz), 1.21 (t, 3H, J = 7.06 Hz), 3.38–3.46 (m, 4H), 3.67 (s, 2H), 4.42–4.52 (m, 4H), 4.73 (s, 2H), 6.95 (s, 3H). ^13^C-NMR (CDCl_3_) δ 12.75, 14.20, 39.75, 40.24, 41.42, 60.62 (q, 1C, J = 36.81 Hz), 67.87 (q, 1C, J = 36.37 Hz), 68.34, 115.09, 118.56, 122.63 (q, 1C, J = 276.92 Hz), 123.11 (q, 1C, J = 277.63 Hz), 124.76, 126.63, 147.34, 148.32, 166.52, 169.76. HRMS (ESI) m/z 446.1359 ([M+H]^+^).

2,2,2-trifluoroethyl 2-(4-(2-(diethylamino)-2-oxoethoxy)-3-methoxyphenyl)acetate (5g): This was synthesized from 4g and 2-chloro-N,N-diethylacetamide (Figure S1, Figure S2, Figure S3, Figure S4). light yellow oil; yeild 78.9%. ^1^H-NMR (CDCl_3_) δ 1.14 (t, 3H, J = 7.13 Hz), 1.21 (t, 3H, J = 7.02 Hz), 3.40–3.45 (m, 4H), 3.67 (s, 2H), 3.88 (s, 3H), 4.46–4.52 (m, 2H, J = 8.42 Hz), 4.74 (s, 2H), 6.78–6.93 (m, 3H). ^13^C-NMR (CDCl_3_) δ 11.73, 13.20, 39.06, 39.27, 40.51, 54.79, 59.46 (q, 1C, J = 36.49 Hz), 67.56, 111.99, 113.32, 120.55, 122.07 (q, 1C, J = 276.44 Hz), 125.63, 146.14, 148.65, 165.95, 169.06. HRMS (ESI) m/z 378.1537 ([M+H]^+^).

3,3,3-trifluoropropyl 2-(4-(2-(diethylamino)-2-oxoethoxy)-3-methoxyphenyl)acetate (5h): This was synthesized from 4h and 2-chloro-N,N-diethylacetamide. light yellow oil; yield: 82.6%. ^1^H-NMR (CDCl_3_) δ 1.14 (t, 3H, J = 7.12 Hz), 1.20 (t, 3H, J = 7.12 Hz), 2.05–2.10 (m, 2H), 3.38–3.46 (m, 4H), 3.72 (s, 2H), 3.87 (s, 3H), 4.04–4.10 (m, 2H), 4.73 (s, 2H), 6.77–6.93 (m, 3H). ^13^C-NMR (CDCl_3_) δ 12.75, 13.43, 14.19, 40.27, 40.47, 41.51, 55.85, 67.14 (q, 1C, *J* = 33.42 Hz), 68.88, 112.94, 114.47, 121.52, 123.89 (q, 1C, *J* = 279.91 Hz), 126.80, 147.07, 149.63, 167.03, 169.87. HRMS (ESI) m/z 392.1683 ([M+H]^+^).

1,1,1-trifluoropropan-2-yl 2-(4-(2-(diethylamino)-2-oxoethoxy)-3-methoxyphenyl)acetate (5i): This was synthesized from 4i and 2-chloro-N,N-diethylacetamide. light yellow oil; yield: 82.6%. ^1^H-NMR (CDCl_3_) δ 1.12 (t, 3H, J = 7.15 Hz), 1.19 (t, 3H, J = 7.15 Hz), 2.40–2.51 (m, 2H), 3.36–3.44 (m, 4H), 3.56 (s, 2H), 3.86 (s, 3H), 4.31(t, 2H, J = 6.34 Hz), 4.72 (s, 2H), 6.75–6.90 (m, 3H). ^13^C-NMR (CDCl_3_) δ 12.76, 14.20, 33.27 (q, 1C, J = 29.06 Hz), 40.27, 40.66, 41.50, 55.81, 57.52 (q, 1C, J = 3.68 Hz), 68.69, 112.94, 114.26, 121.50, 125.71 (q, 1C, J = 277.54 Hz), 127.23, 146.87, 149.49, 167.04, 171.25. HRMS (ESI) m/z 392.1683 ([M+H]^+^).

2,2,2-trifluoroethyl 2-(4-(2-(diethylamino)-2-oxoethoxy)-3-ethoxyphenyl)acetate (5j): This was synthesized from 4j and 2-chloro-N,N-diethylacetamide (Figure S5, Figure S6, Figure S7, Figure S8). light yellow oil; yeild 84.3%. ^1^H-NMR (CDCl_3_) δ 1.11 (t, 3H, J = 7.10 Hz), 1.19 (t, 3H, J = 7.10 Hz), 1.44 (t, 3H, J = 7.01 Hz), 3.38 (m, 4H), 3.62 (s, 2H), 4.03–4.09 (m, 2H), 4.42–4.48 (m, 2H), 4.68 (s, 2H), 6.73–6.89 (m, 3H). ^13^C-NMR (CDCl_3_) δ 11.73, 13.26, 13.79, 39.12, 39.18, 40.55, 54.79, 59.53 (q, 1C, J = 36.72 Hz), 63.33, 67.99, 113.25, 113.86, 117.75, 120.99 (q, 1C, J = 277.30 Hz), 125.53, 146.24, 148.01, 166.08, 169.05. HRMS (ESI) m/z 392.1681 ([M+H]^+^).

3,3,3-trifluoropropyl 2-(4-(2-(diethylamino)-2-oxoethoxy)-3-ethoxyphenyl)acetate (5k): This was synthesized from 4k and 2-chloro-N,N-diethylacetamide. light yellow oil; yield: 78.4%. ^1^H-NMR (CDCl_3_) δ 1.14 (t, 3H, J = 7.14 Hz), 1.22 (t, 3H, *J* = 7.07 Hz), 1.39 (d, 3H, *J* = 6.69 Hz), 2.05–2.12 (m, 2H), 3.38–3.47 (m, 4H), 3.72 (s, 2H), 4.06–4.12 (m, 4H), 4.71 (s, 2H), 6.76–6.91 (m, 3H). ^13^C-NMR (CDCl_3_) δ 12.72, 13.43, 14.25, 14.79, 40.20, 40.44, 41.56, 64.39, 67.11 (q, 1C, *J* = 33.46 Hz), 69.15, 114.40, 115.06, 121.53, 123.89 (q, 1C, *J* = 278.55 Hz), 126.89, 147.27, 149.07, 167.14, 169.89. HRMS (ESI) m/z 406.1875 ([M+H]^+^).

1,1,1-trifluoropropan-2-yl 2-(4-(2-(diethylamino)-2-oxoethoxy)-3-ethoxyphenyl)acetate (5l): This was synthesized from 4l and 2-chloro-N,N-diethylacetamide. light yellow oil; yield: 83.2%. ^1^H-NMR (CDCl_3_) δ 1.13 (t, 3H, J = 7.05 Hz), 1.21 (t, 3H, J = 7.13 Hz), 1.25 (t, 3H, J = 7.02 Hz), 1.43 (t, 3H, J = 7.02 Hz), 3.37–3.46 (m, 4H), 3.52 (s, 2H), 4.06–4.17 (m, 2H), 4.69 (s, 2H), 4.95–5.02 (m, 1H), 6.75–6.91 (m, 3H). ^13^C-NMR (CDCl_3_) δ 12.73, 14.15, 14.26, 14.82, 40.20, 40.94, 41.56, 60.76, 64.38 (q, 1C, J = 35.88 Hz), 69.14, 114.54, 115.06, 121.51, 124.53 (q, 1C, J = 279.64 Hz), 128.12, 146.97, 148.96, 167.22, 171.68. HRMS (ESI) m/z 406.1876 ([M+H]^+^).

Propannidid (Figure S9, Figure S10, Figure S11): This was synthesized from 4m and 2-chloro-N,N-diethylacetamide. light yellow oil; yield: 88.2%. ^1^H-NMR (CDCl_3_) δ 0.92 (t, 3H, J = 7.46 Hz), 1.14 (t, 3H, J = 7.12 Hz), 1.20 (t, 3H, J = 7.07 Hz), 1.61–1.70 (m, 2H), 3.38–3.48 (m, 4H), 3.55 (s, 2H), 3.87(s, 3H), 4.04–4.13 (m, 4H), 4.72 (s, 2H), 6.78–6.93 (m, 3H). ^13^C-NMR (CDCl_3_) δ 10.33, 12.78, 14.22, 21.94, 40.26, 41.01, 41.52, 55.85, 66.43, 68.89, 113.00, 114.33, 121.49, 128.03, 146.74, 149.47, 167.11, 171.78. HRMS (ESI) m/z 338.1969 ([M+H]^+^).

AZD3043 (Figure S12, Figure S13, Figure S14): This was synthesized from 4n and 2-chloro-N,N-diethylacetamide. light yellow oil; yield: 84.6%. ^1^H-NMR (CDCl_3_) δ 0.93 (t, 3H, *J* = 7.43 Hz), 1.15 (t, 3H, *J* = 6.99 Hz), 1.22 (t, 3H, *J* = 6.99 Hz), 1.45 (t, 3H, J = 6.99 Hz), 1.61–1.70 (m, 2H), 3.37–3.46 (m, 4H), 3.56 (s, 2H), 3.87(s, 3H), 4.06 (t, 2H, 6.67 Hz), 4.72 (s, 2H), 6.78–6.92 (m, 3H).^1^H-NMR (CDCl_3_) δ. ^13^C-NMR (CDCl_3_) δ 5.58, 8.01, 9.54, 10.10, 17.19, 35.45, 36.25, 36.83, 59.59, 61.67, 64.45, 109.69, 110.22, 116.76, 123.39, 142.19, 144.18, 162.47, 167.05. HRMS (ESI) m/z 352.2128 ([M+H]^+^).

Propofol (Figure S15, Figure S16, Figure S17): ^1^H-NMR (CDCl_3_) δ 1.35 (s, 6H), 1.37 (s, 6H), 3.20–3.30 (m, 2H), 4.86 (s, 1H), 6.97–7.15 (m, 3H). ^13^C-NMR (CDCl_3_) δ 22.79 (4), 27.22 (2), 120.70, 123.49 (2), 133.74 (2), 150.01. HRMS (ESI) m/z 179.1447 ([M+H]^+^).

### Octanol:Water Partition Coefficients

One mg of each hypnotic was added to 10 ml of water buffered with 10 mm Tris (pH 7.4) and 1 ml of octanol. The mixture was stirred overnight and then centrifuged to fully separate the organic and aqueous phases. The relative hypnotic concentration in each phase (i.e., the partition coefficient) was determined by high-performance liquid chromatography as described for blood.

### Drug Formulation

10 mg/ml emulsion was prepared through the following procedure. Test Compound (1.0% w/v), soybean oil (10.0% w/v), egg phosphatide (1.2% w/v), glycerol (2.5% w/v), oleic acid (0.03% w/v) and water for injection were mixed at 60°C, the pH was adjusted to 8 by 0.1 mol/L NaOH. The mixed solution was stirred with a Polytron tissue homogenizer for 5 min at maximum speed to provide the premixed solution. The premixed solution was circulated through the microfluidizer (12000∼15000 psi) for 30 min to get the final emulsion.

### Ethics Statement

All research involving animals in this study follow the guidelines of the byelaw of experiments on animals, and have been approved by the Ethics and Experimental Animal Committee of Jiangsu Nhwa Pharmaceutical Co., Ltd. The samples were collected and used after obtaining informed consents and approval from the Ethical Committee of Clinical Center of China. All participants provided their written informed consent to participate in this study. The form of informed consent was approved by the Ethical Committee of Clinical Center of China.

### Animals

Chinese Kun Ming mice (male, 20±2.0 g, 6–8 weeks), Sprague-Dawley rats (male, 250±5.0 g, 8 weeks) and New Zealand White rabbits (male, 2.8±3.1 kg, 8–10 weeks) were used. All the animals were purchased from Xuzhou Medical College (Jiangsu, China). The animals were housed under standardized conditions in terms of light and temperature, and received standard rat chow and tap water ad libitum. The animals were randomly assigned to different experimental groups, each kept in a separate cage. All animal research in this study followed the guidelines of the byelaw of experiments on animals, and was approved by the Ethics and Experimental Animal Committee of Jiangsu Nhwa Pharmaceutical Co., Ltd.

### Hypnotic Potency (HD_50_)

All tests were performed during the light period. Groups of mice, rats and rabbits were injected intravenously (lateral tail vein) over 10 s with each dose of test compound, placed in separate boxes to reduce external stimuli and assessed for the loss of righting reflex. Dosing was performed using mg/kg scheme. A set of dose levels was chosen, and depending on whether the loss of righting reflex was observed, extra doses were introduced to provide data for the loss of righting reflex over a narrow dose range to allow potency calculations. From the percentage of mice, rats and rabbits in each group showing loss of righting reflex for ≥30 s, a probit analysis (SAS Institute) was performed to obtain an HD_50_ for each compound with 95% confidence limits.

### The Duration of LORR [15]

Immediately following the injection, the mice, rats and rabbits were tested for loss of righting reflex. If an immediate loss did not occur, the mice were observed closely and placed on their backs to determine the time of the loss of righting reflex. Once a loss was observed, the interval between the loss of righting reflex and the return to righting reflex was recorded. The anesthetized animals recovered righting reflexes spontaneously. To compare the duration of LORR of each compound at equipotent doses, groups of mice, rats and rabbits were injected with each compound at 2× HD_50_ over 10 s.

### Time to Walk

Time to walk was the time required after righting before mice and rabbits walked from the center to the periphery of a 30-cm diameter dise.

### Time to Behavioral Recovery

The rotarod test was conducted to evaluate the recovery to the motor coordination. Before the LORR test, mice were trained for 2 consecutive days. Trained mice could maintain their balance on rod for more than 2 mins. After the mice could walk from the center to the periphery of a 30-cm diameter dise, they were put on the rotarod. Mice were classified as behavioral recovery when, on being placed on the rod, they could maintain their balance for 20 s. The time to behavioral recovery was the time required after mice walked from the centre to the periphery of a 30-cm diameter disc before they could maintain their balance on rod for 20 s.

### Therapeutic Index (TI)

The LD_50_ was determined in mice, rats, and rabbits and the TI was calculated as LD_50_/HD_50_.

### Hypnosis in Response to Intravenous (IV) Infusion [6]

Induction of hypnosis in rabbits was achieved using 2× HD_50_ dose of test compound, and immediately after induction, infusion via the tail vein was commenced at half the HD_50_ dosage per min. the infusion rate was maintained for 20 min or 1 h or 3 h later. The depth of hypnosis was monitored by the magnitude of a withdrawal reflex to intermittent paw pinch provided by a pair of forceps.

### In Vitro Binding Assay [16] [17] [18] [19] [20]

#### General procedures

All the new compounds were dissolved in 5% DMSO. The following specific radioligands and tissue sources were used: (a) GABA_A_ receptor, [^3^H]EBOB, rat frontoparietal cortex; (b) serotonin 5-HT_1A_ receptor, [^3^H]8-OH-DPAT, rat brain cortex; (c) serotonin 5-HT_2A_ receptor, [^3^H]ketanserin, rat brain cortex; (d) serotonin 5-HT_2C_ receptor, [^3^H]mesulergine, rat brain cortex; (e) 5-HTT receptor, [^3^H]paroxetine, rat cerebral cortex; (f) dopamine D_2_ receptor, [^3^H]spiperone, rat striatum; (g) dopamine D_3_ receptor, [^3^H]7-OH-DPAT, rat olfactory tubercle; (h) histamine H_1_ receptor, [^3^H]mepyramine, guinea pig cerebellum; (i) adrenergic α_1_ receptor, [^3^H]prazosin, rat brain cortex; (j) adrenergic α_2_ receptor, [^3^H]rauwolscine, rat brain cortex;

Total binding was determined in the absence of no-specific binding and compounds. Specific binding was determined in the presence of compounds. Non-specific binding was determined as the difference between total and specific binding.

Percentage of inhibition (%)  =  (total binding − specific binding) ×100%/(total binding − nonspecific binding)

Blank experiments were carried out to determine the effect of 5% DMSO on the binding and no effects were observed. Compounds were tested at least three times at 50 µM.

#### GABA_A_ binding assay

Membrane suspensions were thawed and centrifuged in incubation buffer at 10,000×g for 10 min and washed by a similar centrifugation. For displacement studies, membrane suspensions (5 mg original wet wt./ml) were incubated with 0.5 nM [^3^H]EBOB (26.2 µCi/mmol, Perkin Elmer Life Sciences, Boston, MA, USA) in the absence or presence of 50 µM of new compounds or reference drug for 2 h at 25°C. Nonspecific binding was determined in the presence of 10 µM Bicuculline. Bicuculline and picrotoxin were frequently used to determine the nonspecific binding when [3H]EBOB as radioligand [17]. Triplicate 1 ml samples were filtered on Whatman GF/B filters under vacuum with a Brandel Harvester and washed with 3×3 ml ice-cold buffer. Radioactivity bound was measured using a Beckman LS 6500 liquid scintillation counter.

#### 5-HT_1A_ binding assay

Rat cerebral cortex was homogenized in 20 volumes of ice-cold Tris-HCl buffer (50 mM, pH 7.7) using an ULTRA TURAX homogeniser, and was then centrifuged at 32000 g for 10 min. The resulting pellet was then resuspended in the same buffer, incubated for 10 min at 37 °C, and centrifuged at 32000 g for 10 min. The final pellet was resuspended in Tris-HCl buffer containing 10 µM Pargyline, 4 mM CaCl_2_ and 0.1% ascorbic acid. Total binding each assay tube was added 900 µL of the tissue suspension, 50 µL of 0.5 nM [^3^H]8-OH-DPAT (187.4 Ci/mmol, Perkin Elmer Life Sciences, Boston, MA, USA), 50 µL Tris–HCl buffer containing 10 µM Pargyline, 4 mM CaCl_2_ and 0.1% ascorbic acid. Non-specific binding each assay tube was added 900 µL of the tissue suspension, 50 µL of [^3^H] 8-OH-DPAT, 50 µL of 10 µM serotonin. Specific binding each assay tube was added 900 µL of the tissue suspension, 50 µL of [^3^H]8-OH-DPAT, 50 µL of 50 µM new compounds. The tubes were incubated at 37 °C for 30 min. The incubation was followed by a rapid vacuum filtration through Whatman GF/B glass filters, and the filtrates were washed twice with 5 mL cold buffer and transferred to scintillation vials. Scintillation fluid (3.0 mL) was added and the radioactivity bound was measured using a Beckman LS 6500 liquid scintillation counter.

#### 5-HT_2A_ binding assay

Rat cerebral cortex was homogenized in 20 volumes of ice-cold Tris-HCl buffer (50 mM, pH 7.7) using an ULTRA TURAX homogeniser, and centrifuged at 32000 g for 20 min. The resulting pellet was resuspended in the same quantity of the buffer centrifuged for 20 min. The final pellet was resuspended in 50 volumes of the Tris-HCl buffer. Total binding each assay tube was added 900 µL of the tissue suspension, 50 µL of 0.6 nM [^3^H]ketanserine (60.0 Ci/mmol, Perkin Elmer Life Sciences, Boston, MA, USA), 50 µL Tris-HCl buffer. Non-specific binding each assay tube was added 900 µL of the tissue suspension, 50 µL of [^3^H]ketanserin, 50 µL of 10 µM methisergide. Specific binding each assay tube was added 900 µL of the tissue suspension, 50 µL of [^3^H]ketanserin, 150 µL of 50 µM new compounds. The tubes were incubated at 37 °C for 15 min. The incubation was followed by a rapid vacuum filtration through Whatman GF/B glass filters, and the filtrates were washed twice with 5 mL cold buffer and transferred to scintillation vials. Scintillation fluid (3.0 mL) was added and the radioactivity bound was measured using a Beckman LS 6500 liquid scintillation counter.

#### 5-HT_2C_ binding assay

Rat cerebral cortex was homogenized in 20 volumes of ice-cold Tris-HCl buffer (50 mM, pH 7.7) using ULTRA TURAX homogeniser, and centrifuged at 32000 g for 20 min. The resulting pellet was resuspended in the same quantity of the buffer centrifuged for 20 min. The final pellet was resuspended in 50 volumes of the Tris-HCl buffer. Total binding each assay tube was added 900 µL of the tissue suspension, 50 µL of [^3^H]mesulergine, 50 µL Tris-HCl buffer. Non-specific binding each assay tube was added 900 µL of the tissue suspension, 50 µL of 1 nM [^3^H]mesulergine (85.4 Ci/mmol; Perkin Elmer Life Sciences, Boston, MA, USA), 50 µL of 10 µM mianserin. Specific binding each assay tube was added 900 µL of the tissue suspension, 50 µL of [^3^H]mesulergine, 50 µL of 50 µM new compounds. The tubes were incubated at 37 °C for 15 min. The incubation was followed by a rapid vacuum filtration through Whatman GF/B glass filters, and the filtrates were washed twice with 5 mL cold buffer and transferred to scintillation vials. Scintillation fluid (3.0 mL) was added and the radioactivity bound was measured using a Beckman LS 6500 liquid scintillation counter.

#### 5-HTT binding assay

Adult rat cerebral cortex was homogenized in 20 volumes of ice-cold Tris–HCl buffer (50 mM, pH 7.4) using an ULTRA TURAX homogeniser, and was then centrifuged at 20,000 g for 10 min. The resulting pellet was then resuspended in the same buffer, incubated for 10 min at 37 °C, and centrifuged at 32,000 g for 10 min. The final pellet was resuspended in Tris–HCl buffer containing 150 mM NaCl and 5 mM KCl. Total binding each assay tube was added 900 µL of the tissue suspension, 50 µL of 1 nM [3H]paroxetine (22.9 Ci/mmol, Perkin Elmer Life Sciences, Boston, MA, USA), 50 µL Tris–HCl buffer containing 150 mM NaCl and 5 mM KCl. Non-specific binding each assay tube was added 900 µL of the tissue suspension, 50 µL of 1 nM [^3^H]paroxetine, 50 µL of 10 µM paroxetine. Specific binding each assay tube was added 900 µL of the tissue suspension, 50 µL of 1 nM [3H]paroxetine, 50 µL of 50 µM new compounds. The tubes were incubated at 22 °C for 60 min. The incubation was followed by a rapid vacuum filtration through Whatman GF/B glass filters, and the filtrates were washed twice with 5 mL cold buffer and transferred to scintillation vials. Scintillation fluid (3.0 mL) was added and the radioactivity bound was measured using a Beckman LS 6500 liquid scintillation counter.

#### D_2_ binding assay

Rat striatum was homogenized in 20 volumes of ice-cold 50 mM Tris-HCl buffer (pH 7.7) using an ULTRA TURAX homogeniser, and centrifuged twice for 10 min at 48,000 g with resuspension of the pellet in fresh buffer. The final pellet was resuspended in 50 mM ice-cold Tris-HCl containing 120 mM NaCl, 5 mM KCl, 2 mM CaCl_2_, 1 mM MgCl_2_, 0.1% ascorbic acid and 5 µM pargyline. Total binding each assay tube was added 900 µL of the tissue suspension, 50 µL of 0.5 nM [^3^H]spiperone (16.2 Ci/mmol; Perkin Elmer Life Sciences, Boston, MA, USA), 50 µL Tris-HCl buffer containing 120 mM NaCl, 5 mM KCl, 2 mM CaCl_2_, 1 mM MgCl_2_, 0.1% ascorbic acid and 5 µM pargyline. Non-specific binding each assay tube was added 900 µL of the tissue suspension, 50 µL of [^3^H]spiperone, 50 µL of 5 µM (+)-butaclamol. Specific binding each assay tube was added 900 µL of the tissue suspension, 50 µL of [^3^H]spiperone, 50 µL of 50 µM new compounds. The tubes were incubated at 37 °C for 15 min. The incubation was followed by a rapid vacuum filtration through Whatman GF/B glass filters, and the filtrates were washed twice with 5 mL cold buffer and transferred to scintillation vials. Scintillation fluid (3.0 mL) was added and the radioactivity bound was measured using a Beckman LS 6500 liquid scintillation counter.

#### D_3_ binding assay

Rat olfactory tubercle was homogenized in 20 volumes of ice-cold 50 mM Hepes Na (pH 7.5) using an ULTRA TURAX homogeniser, and centrifuged twice for 10 min at 48,000 g with resuspension of the pellet in fresh buffer. The final pellet was resuspended in 50 mM Hepes Na, pH 7.5, containing 1 mM EDTA, 0.005% ascorbic acid, 0.1% albumin, and 200 nM eliprodil. Total binding each assay tube was added 900 µL of membranes, 50 µL of 0.6 nM [^3^H] 7-OH-DPAT (50 Ci/mmol; Perkin Elmer Life Sciences, Boston, MA, USA), 50 µL of 50 mM Hepes Na, pH 7.5, containing 1 mM EDTA, 0.005% ascorbic acid, 0.1% albumin, 200 nM eliprodil. Non-specific binding each assay tube was added 900 µL of membranes, 50 µL of [^3^H] 7-OH-DPAT, 50 µL of 1 µM dopamine. Specific binding each assay tube was added 900 µL of Membranes, 50 µL of [^3^H] 7-OH-DPAT, 50 µL of 50 µM new compounds. The tubes were incubated at 25 °C for 60 min. The incubation was followed by a rapid vacuum filtration through Whatman GF/B glass filters, and the filtrates were washed twice with 5 mL cold buffer and transferred to scintillation vials. Scintillation fluid (3.0 mL) was added and the radioactivity bound was measured using a Beckman LS 6500 liquid scintillation counter.

#### H_1_ binding assay

Guinea pig cerebellum was homogenized in 20 volumes of ice-cold 50 mM phosphate buffer (pH = 7.4) using an ULTRA TURAX homogeniser, and centrifuged twice for 10 min at 50,000 g with resuspension of the pellet in fresh buffer. The final pellet was resuspended in phosphate buffer. Total binding each assay tube was added 900 µL of membranes 50 µL of 1 nM [^3^H] mepyramine (20.0 Ci/mmol; Perkin Elmer Life Sciences, Boston, MA, USA), 50 µL phosphate buffer. Non-specific binding each assay tube was added 900 µL of membranes, 50 µL of [^3^H]mepyramine, 50 µL of 1 µM promethazine. Specific binding each assay tube was added 900 µL of Membranes, 50 µL of [^3^H] mepyramine, 50 µL of 50 µM new compounds. The tubes were incubated at 30 °C for 60 min. The incubation was followed by a rapid vacuum filtration through Whatman GF/B glass filters, and the filtrates were washed twice with 5 mL cold buffer and transferred to scintillation vials. Scintillation fluid (3.0 mL) was added and the radioactivity bound was measured using a Beckman LS 6500 liquid scintillation counter.

#### α_1_ binding assay

Rat cerebral cortex was homogenized in 20 volumes of ice-cold Tris-HCl buffer containing 5 mM EDTA (50 mM, pH 7.7) using ULTRA TURAX homogeniser, and centrifuged at 44000 g for 20 min at 4°C The resulting pellet was resuspended in the same quantity of the buffer centrifuged for 20 min. The final pellet was resuspended in 50 volumes of the Tris-HCl buffer. Total binding each assay tube was added 900 µL of the tissue suspension, 50 µL of 1 nM [^3^H] prazosin (85.4 Ci/mmol; Perkin Elmer Life Sciences, Boston, MA, USA), 50 µL Tris-HCl buffer. Non-specific binding each assay tube was added 900 µL of the tissue suspension, 50 µL of 1 nM [^3^H]prazosin, 50 µL of 10 µM prazosin. Specific binding each assay tube was added 900 µL of the tissue suspension, 50 µL of [^3^H] prazosin, 50 µL of 50 µM new compounds. The tubes were incubated at 25°C for 60 min. The incubation was followed by a rapid vacuum filtration through Whatman GF/B glass filters, and the filtrates were washed twice with 5 mL cold buffer and transferred to scintillation vials. Scintillation fluid (3.0 mL) was added and the radioactivity bound was measured using a Beckman LS 6500 liquid scintillation counter.

#### α_2_ binding assay

Rat cerebral cortex was homogenized in 20 volumes of ice-cold Tris-HCl buffer containing 5 mM EDTA (50 mM, pH 7.7) using ULTRA TURAX homogeniser, and centrifuged at 44000 g for 20 min at 4°C. The resulting pellet was resuspended in the same quantity of the buffer centrifuged for 20 min. The final pellet was resuspended in 50 volumes of the Tris-HCl buffer. Total binding each assay tube was added 900 µL of the tissue suspension, 50 µL of 1 nM [^3^H] rauwolscine, 50 µL Tris-HCl buffer. Non-specific binding each assay tube was added 900 µL of the tissue suspension, 50 µL of 1 nM [^3^H] rauwolscine (73.0 Ci/mmol; Perkin Elmer Life Sciences, Boston, MA, USA), 50 µL of 10 µM rauwolscine. Specific binding each assay tube was added 900 µL of the tissue suspension, 50 µL of [^3^H] rauwolscine, 50 µL of 50 µM new compounds. The tubes were incubated at 25°C for 60 min. The incubation was followed by a rapid vacuum filtration through Whatman GF/B glass filters, and the filtrates were washed twice with 5 mL cold buffer and transferred to scintillation vials. Scintillation fluid (3.0 mL) was added and the radioactivity bound was measured using a Beckman LS 6500 liquid scintillation counter.

### In Vitro GABA_A_ Receptor Electrophysiology [6]

Chinese hamster ovary (CHO)-K1 cells transfected stably with human GABA_A_ receptor α1, β2 and γ2 subunit cDNAs were used. The cells were plated onto collagen-coated 35-mm cell culture dishes at low density for 1–7 days before recording. Test solutions were prepared in the extracellular solution on the day the patch-clamp assay was performed. The intracellular solution consisted of 22 mM KCl, 110 mM K-aspartate, 8 mM NaCl, 1 mM MgCl_2_, 1 mM CaCl_2_, 10 mM ethylene glycol tetraacetic acid, 5 mM Na_2_ATP, 10 mM hydroxyethyl piperazineethanesulfonic acid (HEPES) (adjusted to pH 7.2 using KOH), while the extracellular solution contained 145 mM NaCl, 4 mM KCl, 2 mM CaCl_2_, 2 mM MgCl_2_, 10 mM HEPES (adjusted to pH 7.4 using NaOH). The assay was performed at room temperature. Cells were maintained at −70 mV and the potentiation effect was assessed by co-application of the test compound with EC20 of muscimol after the cell was pre-incubated with the test compound for 45 s. GABA EC20 measured individually in each cell. The amplitude of the Cl− current in response to muscimol and/or test compound was determined. The response to the co-application of the test compound and muscimol was normalized to the control muscimol response as a percentage of the control. A potentiation effect was indicated by a value >100% of the control response. EC50 values were calculated using the four parameter logistic equation, where E was the amplitude of the muscimol-induced current in the presence of the anesthetic at concentration [A], E_MAX_ was the amplitude of the response in the presence of a maximally effective concentration of the anesthetic, EC_50_ was the concentration of anesthetic producing a half-maximal enhancement of the muscimol-induced response and n_H_ was the Hill coefficient.
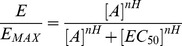



### Measurement of In Vitro Metabolic Half-lives in Blood [21]

Human whole blood stability experiments were conducted in whole blood from four human donors (male, 18–25 years). Single experiments were conducted with pooled blood from Sprague-Dawley rats (male, 8 weeks, n = 3), New Zealand White rabbits (male, 8–10 weeks, n = 3), Beagle dogs (male, 2–3 years, n = 3) and Cynomolgus monkeys (male, 3–4 years, n = 3). All the blood samples were provided by Xuzhou Medical College. A 1.35-ml aliquot of blood was warmed at 37°C for 5 min and hypnotic (from a 40 mM stock in DMSO solution) was added to a final concentration of 200 µM. After the specified incubation time, a 150-µl sample was removed and the metabolic reaction was quenched using 150-µl acetonitrile. Zero time-point samples were prepared by adding 150-µl acetonitrile (using a 4 mM stock in DMSO) to 150-µl blood before addition of the hypnotic. The quenched samples were centrifuged, and the supernatant was separated and stored at −20°C until analyzed. Hypnotic concentrations in thawed samples were determined by high-performance liquid chromatography using a 3-µm phenyl 4.6×50-mm C-A-25, ACE column with the UV detector set at 240 nm. A linear gradient 20–45% acetonitrile in water with 0.05% trifluoroacetic acid was run for more than 20 min at a flow rate of 1 ml/min. The lower limit of quantitation of this assay is 3 µM and the accuracy within 15% at 10 µM. The metabolic half-lives were quantified from the incubation time-dependent change in drug concentration.

### Statistics

In the in vivo hypnotic activity experiments, studies to compare the recovery times of our new compounds, propofol, AZD-3043, and propanidid, a one-way ANOVA with Dunnett's post hoc test (statistical significance at P<0.05) was used. To estimate the potency of test and reference compounds, the ED50 values and their 95% confidence limits were calculated by using the program SPSS (Statistical Package for the Social Science).

## Supporting Information

Figure S1 **^1^H-NMR (400 MHz, CDCl_3_) of Compound 5g.**
(TIF)

Figure S2 **^13^C-NMR (400 MHz, CDCl_3_) of Compound 5g.**
(TIF)

Figure S3 **^19^F-NMR (400 MHz, CDCl_3_) of Compound 5g.**
(TIF)

Figure S4 **HRMS of Compound 5g.**
(TIF)

Figure S5 **^1^H-NMR (400 MHz, CDCl_3_) of Compound 5j.**
(TIF)

Figure S6 **^13^C-NMR (400 MHz, CDCl_3_) of Compound 5j.**
(TIF)

Figure S7 **^19^F-NMR (400 MHz, CDCl_3_) of Compound 5j.**
(TIF)

Figure S8 **HRMS of Compound 5j.**
(TIF)

Figure S9 **^1^H-NMR (400 MHz, CDCl_3_) of Propanidid.**
(TIF)

Figure S10 **^13^C-NMR (400 MHz, CDCl_3_) of Propanidid.**
(TIF)

Figure S11 **HRMS of Propanidid.**
(TIF)

Figure S12 **^1^H-NMR (400 MHz, CDCl_3_) of AZD3043.**
(TIF)

Figure S13 **^13^C-NMR (400 MHz, CDCl_3_) of AZD3043.**
(TIF)

Figure S14 **HRMS of AZD3043.**
(TIF)

Figure S15 **^1^H-NMR (400 MHz, CDCl_3_) of Propofol.**
(TIF)

Figure S16 **^13^C-NMR (400 MHz, CDCl_3_) of Propofol.**
(TIF)

Figure S17 **HRMS of Propofol.**
(TIF)
