# Supplementary figures and images for: Synthesis and Evaluation of Fluorine-Substituted Phenyl Acetate Derivatives as Ultra-Short Recovery Sedative/Hypnotic Agents
Source: PLoS One. 2014 May 5;9(5):e96518. doi: 10.1371/journal.pone.0096518 (PMC4010497; doi:10.1371/journal.pone.0096518)

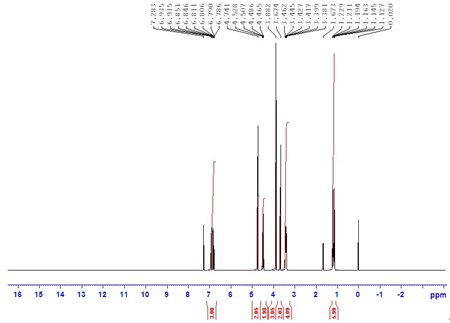

Supplement: Figure S1 — 1H-NMR (400 MHz, CDCl3) of Compound 5g. (TIF) [file pone.0096518.s001.tif]

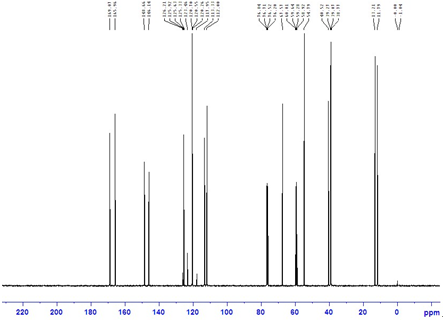

Supplement: Figure S2 — 13C-NMR (400 MHz, CDCl3) of Compound 5g. (TIF) [file pone.0096518.s002.tif]

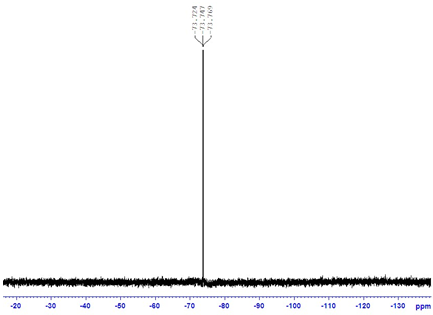

Supplement: Figure S3 — 19F-NMR (400 MHz, CDCl3) of Compound 5g. (TIF) [file pone.0096518.s003.tif]

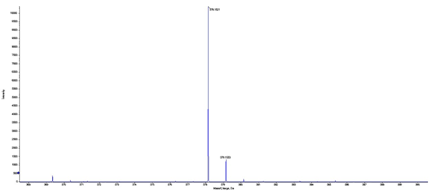

Supplement: Figure S4 — HRMS of Compound 5g. (TIF) [file pone.0096518.s004.tif]

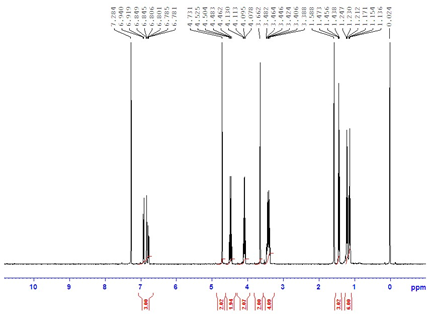

Supplement: Figure S5 — 1H-NMR (400 MHz, CDCl3) of Compound 5j. (TIF) [file pone.0096518.s005.tif]

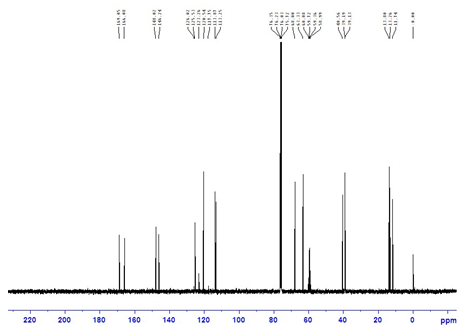

Supplement: Figure S6 — 13C-NMR (400 MHz, CDCl3) of Compound 5j. (TIF) [file pone.0096518.s006.tif]

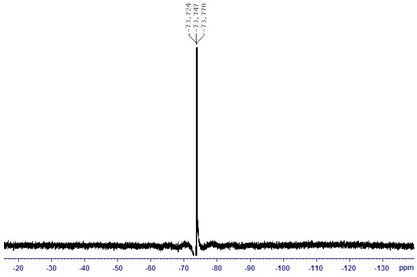

Supplement: Figure S7 — 19F-NMR (400 MHz, CDCl3) of Compound 5j. (TIF) [file pone.0096518.s007.tif]

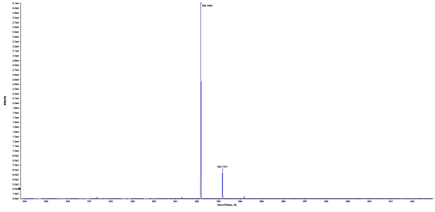

Supplement: Figure S8 — HRMS of Compound 5j. (TIF) [file pone.0096518.s008.tif]

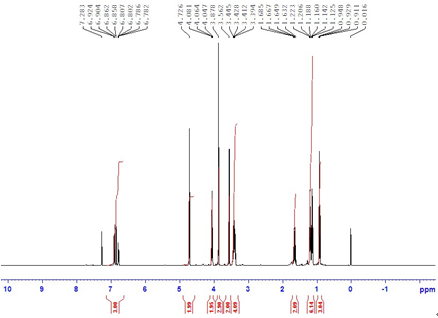

Supplement: Figure S9 — 1H-NMR (400 MHz, CDCl3) of Propanidid. (TIF) [file pone.0096518.s009.tif]

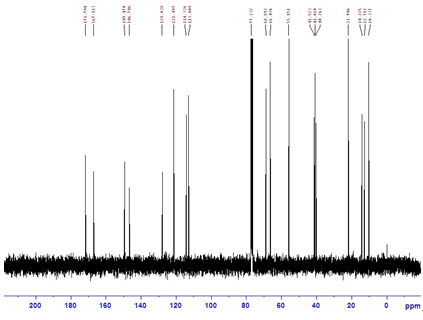

Supplement: Figure S10 — 13C-NMR (400 MHz, CDCl3) of Propanidid. (TIF) [file pone.0096518.s010.tif]

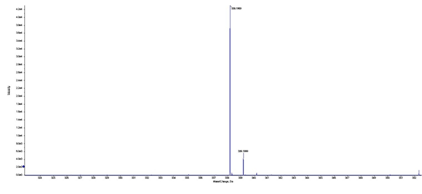

Supplement: Figure S11 — HRMS of Propanidid. (TIF) [file pone.0096518.s011.tif]

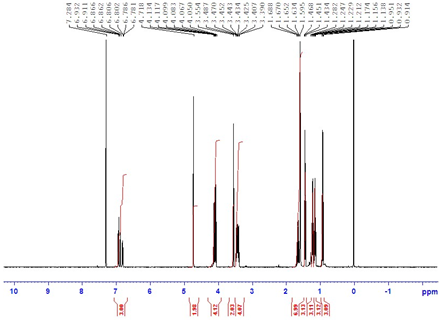

Supplement: Figure S12 — 1H-NMR (400 MHz, CDCl3) of AZD3043. (TIF) [file pone.0096518.s012.tif]

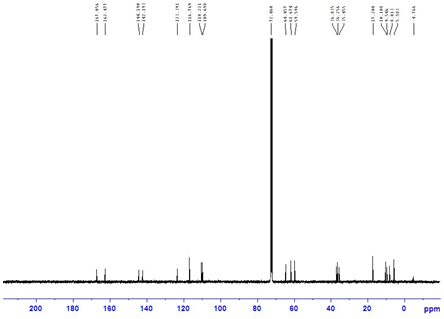

Supplement: Figure S13 — 13C-NMR (400 MHz, CDCl3) of AZD3043. (TIF) [file pone.0096518.s013.tif]

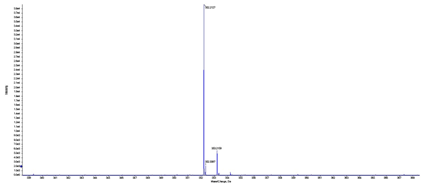

Supplement: Figure S14 — HRMS of AZD3043. (TIF) [file pone.0096518.s014.tif]

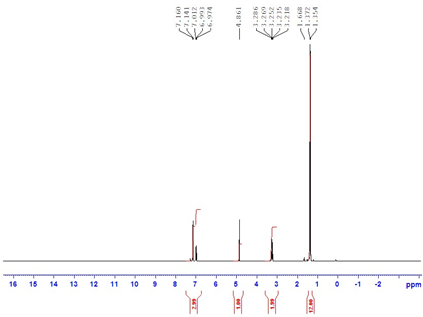

Supplement: Figure S15 — 1H-NMR (400 MHz, CDCl3) of Propofol. (TIF) [file pone.0096518.s015.tif]

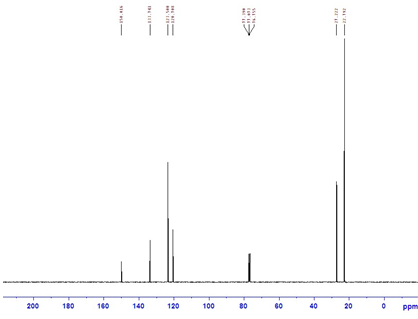

Supplement: Figure S16 — 13C-NMR (400 MHz, CDCl3) of Propofol. (TIF) [file pone.0096518.s016.tif]

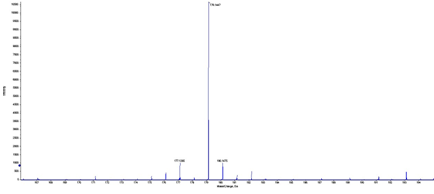

Supplement: Figure S17 — HRMS of Propofol. (TIF) [file pone.0096518.s017.tif]
